# Recent advances in synthetic strategies and SAR of thiazolidin-4-one containing molecules in cancer therapeutics

**DOI:** 10.1007/s10555-023-10106-1

**Published:** 2023-05-19

**Authors:** Archana Sharma, Diksha Sharma, Neha Saini, Sunil V. Sharma, Vijay Kumar Thakur, Ramesh K. Goyal, Prabodh Chander Sharma

**Affiliations:** 1 DIPSAR, Delhi Pharmaceutical Sciences and Research University, New Delhi, 110017 India; 2 Swami Devi Dayal College of Pharmacy, Barwala, 134118 India; 3 School of Chemistry, North Haugh, University of St Andrews, St Andrews, Fife, 16 9ST KYScotland UK; 4 Biorefining and Advanced Materials Research Center, Scotland’s Rural College (SRUC), King’s Buildings, West Mains Road, Edinburgh, EH9 3JG UK; 5 School of Engineering, University of Petroleum & Energy Studies (UPES), Dehradun, 248007 Uttarakhand India; 6 SPS, Delhi Pharmaceutical Sciences and Research University, New Delhi, 110017 India

**Keywords:** Thiazolidin-4-one, Anticancer, Anti-proliferative, Cytotoxic, Anti-tumor, Synthesis, Cell lines

## Abstract

Cancer is one of the life-threatening diseases accountable for millions of demises globally. The inadequate effectiveness of the existing chemotherapy and its harmful effects has resulted in the necessity of developing innovative anticancer agents. Thiazolidin-4-one scaffold is among the most important chemical skeletons that illustrate anticancer activity. Thiazolidin-4-one derivatives have been the subject of extensive research and current scientific literature reveals that these compounds have shown significant anticancer activities. This manuscript is an earnest attempt to review novel thiazolidin-4-one derivatives demonstrating considerable potential as anticancer agents along with a brief discussion of medicinal chemistry-related aspects of these compounds and structural activity relationship studies in order to develop possible multi-target enzyme inhibitors. Most recently, various synthetic strategies have been developed by researchers to get various thiazolidin-4-one derivatives. In this review, the authors highlight the various synthetic, green, and nanomaterial-based synthesis routes of thiazolidin-4-ones as well as their role in anticancer activity by inhibition of various enzymes and cell lines. The detailed description of the existing modern standards in the field presented in this article may be interesting and beneficial to the scientists for further exploration of these heterocyclic compounds as possible anticancer agents.

## Introduction

### Overview of cancer

**Fig. 1:**
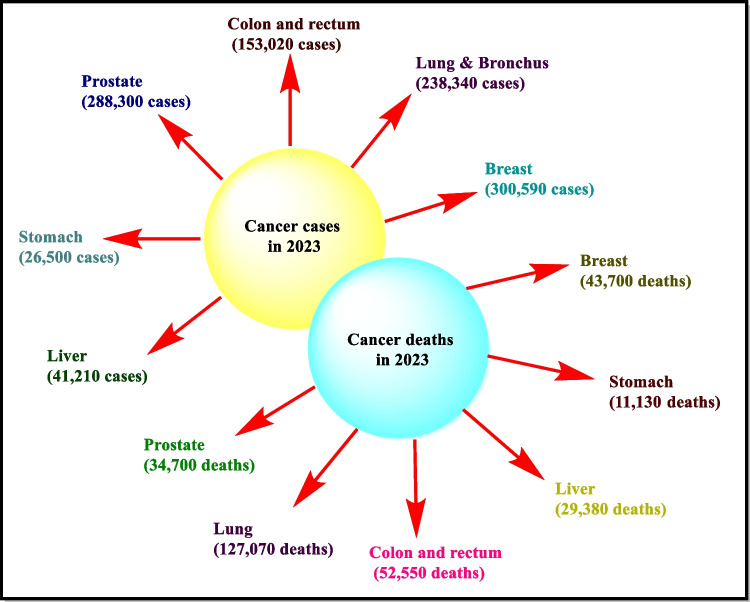
US prediction about new cancer cases and deaths in 2023 (https://cancerstatisticscenter.cancer.org/module/BmVYeqHT)

Cancer is still a global health concern, and it is the second leading cause of death in developed countries [1]. Cancer is a markedly multigenic, complex, multi-factorial, and distinct pathway ailment. A very common characteristic associated with cancer is the fast generation of a group of cells that grow at a rapid rate beyond their limit, invading into adjacent body parts and spreading to the other surrounding organs leading to their destruction and sometimes metastasis [2, 3]. The majority of the cancers result in a tumor, except some such as leukemia, which does not form a tumor. Cancer can occur at any age, including even the fetuses, but the danger for furthermost types of cancer surges with age [4]. On the other hand, benign tumors do not display malignant properties and are generally self-limited and do not show invasion or metastasis of adjacent tissues and organs [5, 6]. It is particular to note that, although the rate of total incidences of cancer observed in the developed countries is double to that of reported in the developing world, the overall death rates in both sexes related to cancer are almost similar. The most frequently spotted and the prime cause for cancer demises in females is breast cancer that accounts for 23% of the entire cancer cases as well as the death of 14% patients annually, whereas the lung is the most likely cancer spot between males responsible for 17% of the entire new cases. From the literature survey, it was estimated that in 2030 the new cases of cancer diagnosis are anticipated to be 21 million around the globe with per year deaths of 17 million people and estimation of people breathing with cancer would be 75 million [7]. Cancer is a leading cause of death worldwide, accounting for nearly 1,958,310 new cancer cases, and 609,820 cancer deaths are anticipated in the USA in 2023 (https://cancerstatisticscenter.cancer.org/module/BmVYeqHT) . The progression of cytotoxicity begins from normal cells of the body. According to researchers, it may develop due to changes in control mechanism by activation of oncogenes and the inconsiderable role of cancer suppressor genes, and may be due to factors responsible for mutagenesis, i.e., due to mutations of genes [8]. As one of the cytoskeleton’s main components, microtubules play a noteworthy role in a number of biological processes, namely the protection of cell shape, molecular signaling pathway, contributing to the movement of cell organelles, and more importing the antly, producing the mitotic spindle to ensure the cell cycle progression [9]. Glioblastoma multiforme (GBM) is the most prevalent primary brain tumor in adults and described by its extremely invasive potential and heterogeneity, which results in chemical resistance and rapid tumor reoccurrence [10]. Many tumor-related deaths in patients under 35 years of age have been regarded as caused by brain cancer. The numerous forms of brain tumors include meningeal, tumors of the cellular region, neuroepithelial, metastatic, and primary CNS lymphomas [11]. Tumor microenvironment has a crucial role in cancer development and metastasis. Macrophages are well-defined inflammation and host defense elements of the effector cells against tumors. Based on the tumor microenvironment, tumor-associated macrophages differentiate into either cytotoxic (M1) or tumor-promoting (M2) phenotypes [12]. Various efforts have been made by scientists for the treatment of malignancy; to fulfill this purpose, different treatment strategies (Fig. 1) like surgery, radiotherapy, hormone therapy, and chemotherapy were used [5]. Among all the treatment options, chemotherapy is the vital and chief treatment option involving natural and synthetic agents that are potent and widely used as anticancer agents [13, 14]. Chemotherapy also has some limitations like severe side effects, toxicity, resistance, and shortage of chemotherapeutic agents. This problem can be solved by continuous research and the growth of innovative chemotherapeutic mediators. For the development of new drugs, DNA is an effective target among all the targets because drugs easily bind to the DNA and unnaturally alter or prevent its functions. The action of enzymes that metabolize DNA like topoisomerase can be repressed by the introduction of apoptosis when drugs bind to DNA and protein structure [13, 15].

Even though ongoing research projects have led to considerable improvements in cancer treatments over the past few decades, numerous research projects are still needed for the efficient management of cancer and its associated symptoms. Moreover, the occurrence of adverse events associated with cancer treatment is imposing another challenge to the medicinal researchers. The major methodology that is primarily used to treat cancer patients depends upon the explicit stage, type, and location [16].

### Physicochemical parameters and applications of nanotechnology for enhancing anticancer activity

The low water solubility of anticancer drugs was a prominent and serious problem that prevented their production and clinical application; many extremely active and effective chemotherapeutic agents were removed due to their low water solubility. A major challenge for the development of pharmaceutical drugs is to eradicate or at least minimize the negative, harmful effects of highly active drugs, especially those antitumor agents that show general toxicity in the body, which significantly limits their use [17]. Nanoparticles (NPs) can maximize the cellular penetration of chemotherapeutic drugs in malignant cells to reduce toxicity by the usage of active and passive target-specific approaches of cells [18]. Nanocarriers found use in separation, enrichment, cancer diagnosis, and cancer therapy [19]. Nanocarriers are encouraging alternatives used extensively in the field of oncology. Furthermore, these drug-charged nanomaterials display several important advantages such as passive targeting of malignant cells by increased permeability and retention effect, enhanced bioavailability, extended diffusion period, and decreased adverse reactions [20]. Nanotechnology being used in different biomedical applications, including the distribution of medications, has drawn growing concern owing to their capacity to change the pharmacokinetics of medicines. Nanomedicines enhance solubility of poorly soluble medications and decrease its metabolism by degradation of their hydrophilic or hydrophobic sections. Nanomedicines have extended half-life in plasma and varying biodistribution levels as compared to modern chemotherapy [21].

**Fig. 2:**
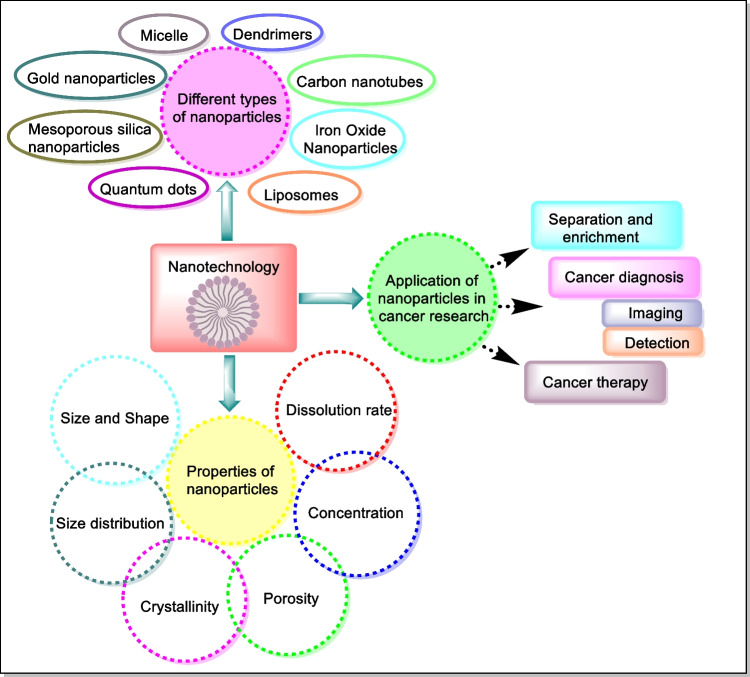
Applications, properties, and different types of nanoparticles in cancer therapy

Physicochemical properties of nanoparticles such as size, shape, size distribution, crystallinity, porosity, concentration, and dissolution rate are predominantly relevant to establish their biological interactions and impacts [22, 23]. Nanoparticles used as drug delivery systems are submicron-sized particles (3–200 nm), structures, or systems that can be made from a range of materials like polymers (polymeric nanoparticles, micelles, or dendrimers), lipids (liposomes), viruses (viral nanoparticles), metallic (gold nanoparticles), oxides (iron oxide nanoparticles), and even organometallic compounds (nanotubes) [24]. Depending on the type of nanoparticles, drugs are released from nanoparticles at a particular concentration via internal or external stimuli. The nanoparticles utilized for the transportation of other substances at a particular site are called nanocarriers. Conventional nanoparticles do not have the ability to transport and release drugs at the right concentration at a specific site. Therefore, they requisite to be altered and functionalized to make them smart [25]. Diverse applications, properties, and types of nanoparticles for cancer treatment are outlined in Fig. 2.

### Thiazolidin-4-one: a brief overview

**Fig. 3:**
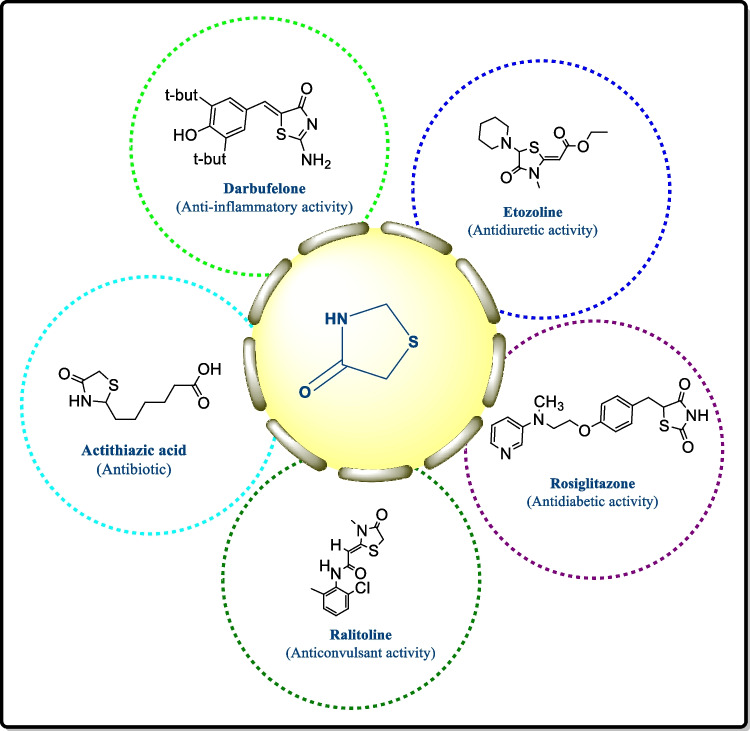
Pharmacological activities of thiazolidin-4-one derivative [55–57]

Modern scientific methods in medicinal chemistry have been employed in association with active pharmacophores in their scientific exploration for improved biological actions to generate highly potent compounds [26]. Heterocyclic compounds bearing nitrogen, sulfur, and thiazole moieties occupy a pivotal place among biologically active substances, so the progress of simple and effective methods for synthesis of these analogues possessing many heterocyclic scaffolds has given a new direction to the modern medicinal chemistry-based drug discovery [27]. Developing novel chemotherapeutic agents is an exigent task for medicinal researchers [28, 29]. In the past few decades, advancement occurred in this field and many antitumor compounds were discovered which were of natural and synthetic origin [30]. Thiazolidin-4-one derivatives play a chief role in contemporary medicinal chemistry as they display a diversity of biological activity [31], i.e., anti-cancer [32–34], anti-convulsant [35], antitubercular [36, 37], anti-microbial [38, 39], anti-diabetic [40], analgesic [41–43], antiparasitic [44, 45], anti-HIV [46, 47], antioxidant [48, 49], anti-malarial [50, 51], COX inhibitory [52, 53], and anti-hypertensive activity [54].The different pharmacological activities performed by thiazolidin-4-one are designed in Fig. 3.

Thiazolidin-4-one is an effective scaffold in the production of drugs that inhibit cancer cell proliferation [58, 59]. Thiazolidin-4-one–containing innovative drugs were established comprising hypoglycemic thiazolidinones (pioglitazone along with its analogues), darbufelon (dual COX-2/5-LOX inhibitors), etozolin (novel diuretics), etc. [60–62].

Thiazolidin-4-one ring systems are also found in the natural products for the treatment of cancer [63–66]. Anticancer agents are classified as DNA-interacting agents, agents of molecular targeting, agents of anti-tubulin, antimetabolites, monoclonal antibodies, hormones, and other biological agents, which proved to be a valuable core to explore newer anticancer medications [67, 68]. The majority of the currently available chemotherapeutic anticancer drugs act either by direct interaction with DNA or by inhibiting the relaxation process of DNA [69, 70]. The quantitative structure–activity relationships based protocols have been developed for thiazolidin-4-ones with objectives to develop more structurally diverse compounds of this class having the desired biological actions [71–73].

### Structure-related aspects

**Fig. 4:**
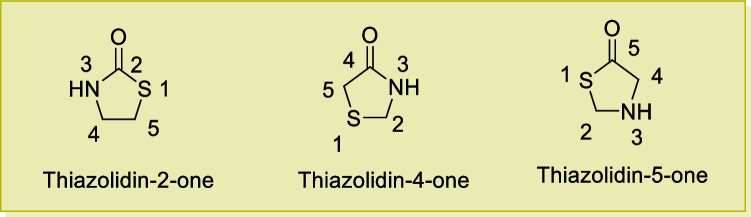
Different isomeric forms of thiazolidinones

Various isomeric thiazolidinones with numbering and nomenclature are depicted in Fig. 4. Thiazolidin-4-one core is widely represented in compounds with diverse biological activities [74–76].

In several literatures, the noncyclic chemical structures for pseudo-thiohydantoin and rhodanine were first proposed because there was considerable confusion regarding the structure of thiazolidin-4-ones [59]. The carbon atom of methylene at site 5 of thiazolidin-4-one analogues depicts nucleophilic action and the Knoevenagel reaction of this (methylene group) has been widely attempted [77]. The carbonyl group of thiazolidin-4-one analogues has been observed to be highly unreactive. However, in some cases, it has been observed that the reaction of thiazolidine-4-ones with Lawesson’s reagent produces the analogous 4-thione analogues [59].

The structural and conformational features of thiazolidin-4-ones are very important to establish a correlation between their biological actions and basicity. Several optical, geometrical, and regioselective isomers of thiazolidin-4-one derivatives have been described and different configurations with the envelope or half-chair conformation have also been observed [73, 78].

### Physico-chemical characteristics

Thiazolidin-4-one substituted at position 3 is a generally solid compound that often melts with decomposition. Their melting point is lowered with the alkyl substitution at the nitrogen atom. Thiazolidinones are slightly soluble in water but when there is no substitution of aryl or a higher alkyl group, they show solubility somewhat [56, 59].

## Synthetic approach

New synthetic strategies reported for development of novel thiazolidin-4-one containing compounds with special emphasis of synthesis using different reagents, click chemistry/green chemistry, and usage of nanocarrier-based catalysts have also been discussed in the following sections.

### Synthetic aspects of thiazolidin-4-one

**Fig. 5:**
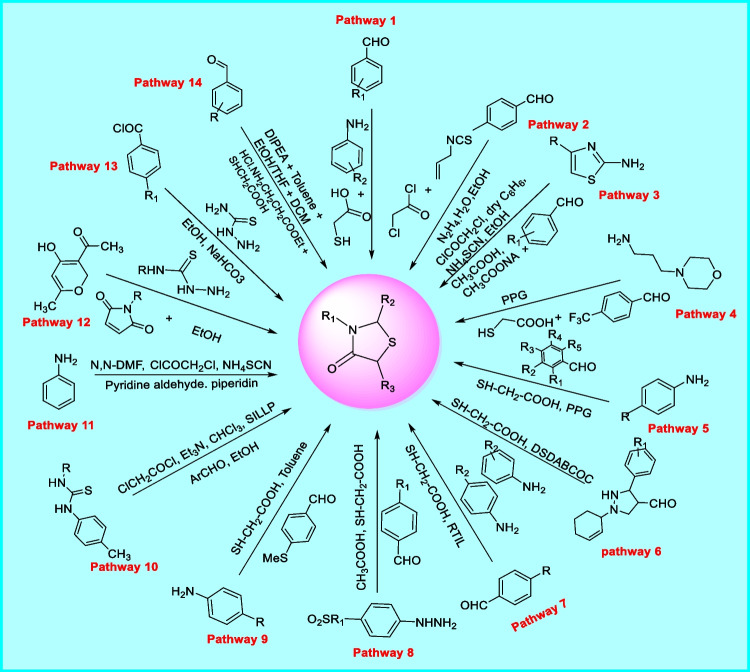
Different synthetic routes for the production of thiazolidin-4-one derivatives

Thiazolidin-4-one heterocycles are also used for the designing of various novel drugs that act at various targets. Some important synthetic schemes (synthetic routes 1–14) are summarized in Fig. 5 [79].

*In synthetic route 1*, Foroughifar et al. designed a one-pot synthesis of 1, 3-thiazolidin-4-ones. In this path, the reaction of aromatic amine, aromatic aldehyde, mercaptoacetic acid, and Bi(SCH_2_COOH)_3_ as a catalyst was done underneath solvent-free conditions. Thin-layer chromatography (TLC) was used to test the completion of reaction and purity of the chemicals. It was also reported that the yield of the product was enhanced by raising the temperature up to 70ºC [80]. *In synthetic route 2*, Kaboudin et al. recognized a sequence of thiazolidine-4-ones and 3*H*-thiazole analogues by means of one-pot four-component condensation-cyclization reaction. In this path, synthesis of thiazolidin-4-one analogues was performed using refluxing hydrazine carbothiomide derivatives and DMAD (dimethyl acetylenedicarboxylate) in 40 ml absolute ethanol with elimination of MeOH [81]. *In synthetic route 3*, Apostolidis et al. synthesized an innovative sequence of 5-arylidene-2-(1, 3-thiazol2-ylimino)-1, 3-thiazolidin-4-ones. In this path, substituted thiazole-2-amine was reacted with α-chloro acetyl chloride, dry benzene, and ammonium thiocyanate to create intermediates. Furthermore, this intermediate is reacted with substituted benzaldehyde, acetic acid, and sodium acetate to obtain thiazolidin-4-ones [82]. *In synthetic route 4*, Prasad et al. prepared an innovative sequence of 2-aryl-thiazolidin-4-one derivatives. In this pathway, aniline, benzaldehyde, and thioglycolic acid were reacted at 110ºC in PPG (polypropylene glycol) and the product was obtained in good yield (83%). Furthermore, all the derivatives synthesized by this route were decontaminated by column chromatography using silica gel of 60–120 mesh. It was reported that even after 24 h no product formation was detected when PEG (polyethylene glycol) is used as a catalyst. This problem was solved by using PPG in place of PEG [83]. *In synthetic route 5*, Prasad et al. prepared an innovative sequence of 2-aryl-thiazolidin-4-one analogues. In this path, the compounds containing the thiazolidin-4-one nucleus were synthesized similarly as synthesized in route 4. All the reactants are similar except the aniline derivative. As already shown thiazolidin-4-one analogues were synthesized in good yield by reacting aniline, aldehyde, and thioglycolic acid at 110ºC in PPG [83]. *In synthetic route 6*, Taherkhorsand et al. planned a sequence of 2-pyrazolo-3-phenyl-1, 3-thiazolidine-4-ones. In this pathway, pyrazole carbaldehyde, anilines, and thioglycolic acid DSDABCOC were reacted to obtain thiazolidin-4-one derivatives. It was found that the reaction gave the product in good yield (82–92%) when DSDABCOC was used as a catalyst. It was also found that the rate of reaction could be raised by using ultrasound and the consumption of energy could be reduced [84]. *In synthetic route 7*, Khillare et al. testified a novel sequence of thiazole-substituted pyrazolyl-4-thiazolidinones. In this path, a reaction between aromatic carbaldehyde, aromatic amines, and mercapto acetic acid in DTPEAC (diisopropyl ethyl ammonium acetate) at room temperature gives a product containing thiazolidin-4-one nucleus [85]. *In synthetic route 8*, Unsal-Tan et al. investigated a novel sequence of 2-aryl-3-(4-sulfamoyl/methylsulfonylphenylamino)-4-thiazolidinones. In this pathway, phenylhydrazine was dissolved with benzaldehyde in methanol and then acetic acid has been incorporated as a catalytic medium in the mixture of reaction and then heated with Dean-stark separator to form an intermediate. Furthermore, the intermediate was refluxed with acid, i.e., mercaptoacetic acid to obtain the urged product [86]. *In synthetic route 9*, Zarghi et al. developed a new sequence of 2, 3-diaryl-1, 3-thiazolidine-4-one analogues. In this path, a reaction of aromatic amine, 4-methyl thiobenzaldehyde, and thioglycolic acid in dry toluene gives an intermediate. Furthermore, oxidation of intermediate with 30% H_2_O_2_ (hydrogen peroxide) in the presence of WO_3_ (tungsten trioxide) gives derivatives of thiazolidin-4-one [87]. *In synthetic route 10*, Jourshari et al. arranged an innovative sequence of 5-arylidene-2-imino-4-thiazolidinone analogues. In this pathway, N′-ethyl-N′-(*P*-tolyl/thiourea) was reacted with the solution of chloroacetyl chloride and chloroform to obtain the product. The solution of chloroacetyl chloride and chloroform was added dropwise [88]. *In synthetic route 11*, Ranga et al. introduced a novel sequence of pyridine analogues of 4-thiazolidinone. In this path, a reaction of “2-chloro-N-arylacetamide” (obtained by aromatic amine, DMF, chloroacetyl chloride reaction) and ammonium thoicyanate in ethanol yields an intermediate. Furthermore, the intermediate was reacted with pyridine aldehyde and piperidine to obtain the product with thiazolidin-4-one nucleus [89]. *In synthetic route 12*, Sadou et al. reconnoitered innovative analogues of 4-thiazolidinone. In this pathway, the reaction of thiosemicarbazide with dehydroacetic acid gives thiosemicarbazenes which further reacted with (ethyl 2-bromo propionate), phenyl bromoacetate, or maleimide to obtain compounds with thiazolidin-4-one moiety [90]. *In synthetic route 13*, Küçükgüzel et al. described a novel sequence of 2-heteroarylimino-5-arylidene-4-thiazolidinone analogues. In this path, thiosemicarbazide was reacted with substituted benzoyl chloride, sodium bicarbonate, aldehyde, and ethanol to obtain thiazolidine-4-one derivatives [91]. *In synthetic route 14*, Apotrosoaei et al. testified a new series of analogues of 4-thiazolidinone. In this path, substituted aromatic aldehyde, ethyl 3-aminopropionate hydrochloride, mercaptoacetic acid, DIPEA (N, N-disopropyl ethylamine), toluene, ethanol, tetrahydrofuran, and dichloromethane were reacted to yield thiazolidine-4-one derivatives [92].

### Click chemistry/green synthesis

**Fig. 6:**
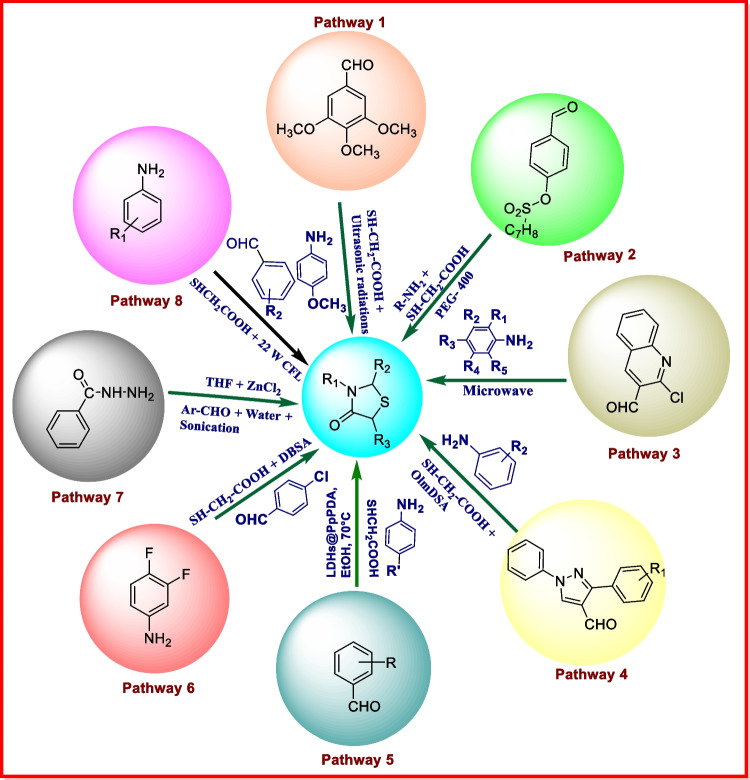
Some examples of green synthesis of thiazolidin-4-one derivatives

The title role of chemistry is vital to ensure that our upcoming materials, energy production, and chemicals are more sustainable as compared to the present generation. The demand of eco-friendly chemical processes and products around the world needs progress of new and cost-effective methods for the prevention of pollution. Moreover, due to the pervasive use of heterocyclic molecules, numerous classical named reactions have been developed in the past to create heterocyclic derivatives. These reactions require thermal heating, harmful or expensive catalysts, high boiling solvents, and prolonged reaction times. When it comes to environmental safety protocols, these strategies are no longer a force to be reckoned in terms of safety. The most effective idea in chemistry to maintain sustainability is green chemistry with an approach towards the use of effective, safe, and efficient chemical methodologies for the synthesis of biologically active compounds. These sustainable methods continue to be in high demand and reduce or eliminate the use of hazardous materials in the context of environmental safety [93, 94]. Several green chemistry–oriented methods include the usage of sonochemistry, ionic liquids, microwave-assisted technology, phase transfer catalysis, and numerous other techniques [95, 96]. The sustainable techniques for synthesis have incredible feature such as lower energy consumption, increase selectivity, proper utilization of raw material and catalyst in the reaction, and less use of harmful solvents. Some green synthesis pathways of thiazolidin-4-ones are detailed in Fig. 6.

*In synthesis 1*, Angapelly et al. described a novel sequence of thiazolidin-4-one derivatives by using the green chemistry protocol. In this pathway, aldehydes, amines, and thioglycolic acid were reacted in the presence of VOSO_4_ (vanadyl sulfate) in acetonitrile under ultrasonic irradiation [97]. *In synthesis 2*, Bhosle et al. arranged a novel sequence of 2, 3-disubstituted-4-thiazolidinones. In this path, the reaction of 4-(*p*-toulysulfonoxy) benzaldehyde, aryl/heteryl amines along with mercaptoacetic acid in PEG-400 gives thiazolidin-4-one derivatives [98]. *In synthesis 3*, Tiwari et al. presented a novel sequence of 2-(2-chloroquinoline-3-yl)-3-substituted phenyl thiazolidin-4-ones. In this path, 2-chloroquinoline-3-carbaldehyde and aromatic aniline condensed under microwave irradiation at 200 W give intermediate N-aryl-2-chloroquinolin-3-yl-azomethine. Furthermore, the intermediate formed was reacted with thioacetic acid (HSCH_2_COOH) and zeolite 5A under microwave irradiation to obtain the desired product [99]. *In synthesis 4*, Nikpassand et al. prepared a novel sequence of 2-pyrazolyl-1, 3- thiazolidine-4-ones using 2-oxoimidazolidine-1, 3-disulfonic acid. In this path, by the reaction of pyrazolcarbaldehyde, aniline and thioglycolic acid were reacted in the presence of mMWCNT nanocomposite and thiazolidin-4-one derivatives were obtained [100]. *In synthesis 5*, Mirzaei-Mosbat et al. introduced an innovative sequence of 1, 3-thiazolidin-4-ones. In this pathway, benzaldehyde, substituted aniline, and thioglycolic acid were reacted in the presence of LDHs@PpPDA catalyst and thiazolidin-4-one derivatives were obtained [101]. *In synthesis 6*, Prasad et al. developed a novel sequence of 2, 3-disubstituted 4-thiazolidinones. In this path, a solution of DBSA (4-dodecylbenzenesulfonic acid) is prepared in water and then aromatic/aliphatic primary amine, thioglycolic acid, and aromatic aldehyde were combined to obtain the desired product [102]. *In synthesis 7*, Thomas et al. used ultrasound to promote Schiff’s base formation of isoniazid with aromatic aldehydes in water, which were converted to corresponding thiazolidinones using thioglycolic acid and zincchloride in THF (tetrahydrofuran) [96]. *In synthesis 8*, in this path, Nazeef et al. explored a new sequence of 1, 3-thiazolidin-4-ones. On a magnetic stirrer, a solution of aniline, thioglycolic acid, and aromatic aldehyde was stirred in a round bottom flask. At room temperature under the exposure of 22-W CFL, the reaction mixture was irradiated. In the round bottom flask, the reaction blend was kept 9 cm away from the compact fluorescent lamp and the reaction growth was observed by TLC [103]. Some green synthetic pathways of thiazolidin-4-ones are detailed in Fig. 6.

### Synthesis of thiazolidinone-based nanomaterials

To overcome difficulties and to speed up the synthesis of nanoparticles, various scientists focused on the development of thiazolidinone-based nanocarriers [104].

**Fig. 7:**
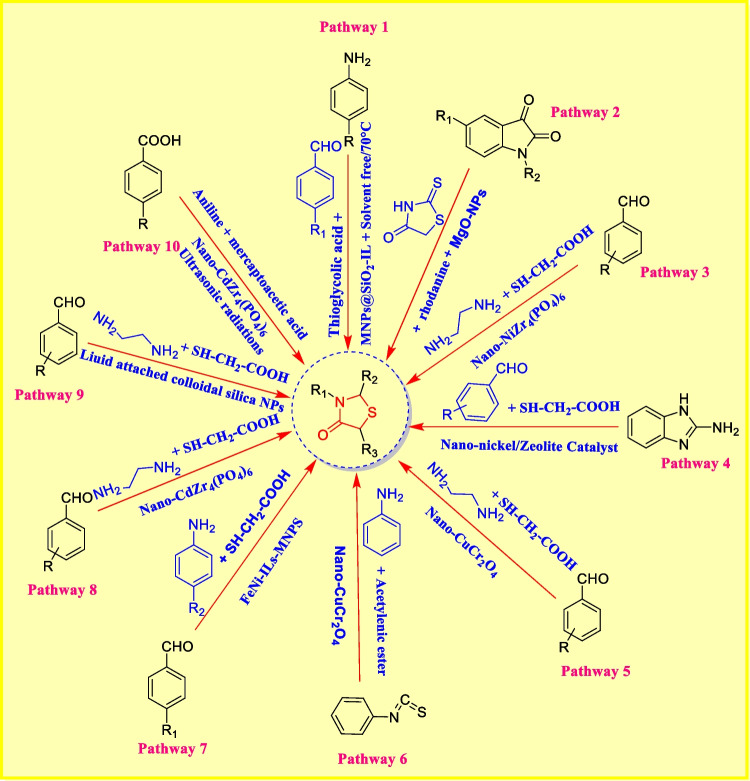
Few examples of production of thiazolidin-4-one derivatives via nanoparticle-based catalysts

Currently, nanoparticles particularly magnetic nanoparticles have fascinated growing attention due to their specific physical properties involving super paramagnetism, high surface area, toxicity, and possible uses in different fields [105]. Scientists have concentrated on the production of nanoparticles over the past decades. Nanotechnology advancement has encouraged scientists to develop and exemplify materials with remarkable properties and nanometric measurements [106]. Here, we discussed some thiazolidinone-based nanomaterials (Fig. 7).

*In pathway 1*, Azgomi et al. described one-pot synthesis of analogues of 1, 3-thiazolidin-4-one. In this path, these analogues were produced by stirring solution of aromatic aldehyde, MNPs@SiO_2_-IL, thioglycolic acid, and anilines at 70 °C for the suitable time [107]. *In pathway 2*, Baharfar et al. presented a new sequence of (isatin-based) 2-amino thiazol-4-ones utilizing (MgO) nanoparticles in aqueous medium. In this reaction, solutions of rhodanine, amine, isatin analogues, and water were (stirred at room temperature) in the presence of magnesium oxide nanoparticles [108]. *In pathway 3*, Safaei-ghomi et al. investigated bis-thiazolidinones catalyzed by nano-NiZr_4_ (PO_4_)_6_ under microwave irradiation. A solution of aldehyde, thioglycolic acid, ethylenediamine, and nano-NiZr_4_ (PO_4_)_6_ in toluene was exposed to microwave irradiation fora specific time [109]. *In pathway 4*, Kalhor et al. introduced a new sequence of 3-benzimidazolyl-4-thiazolidinone analogues. A solution of 2-aminobenzimidazole, thioglycolic acid, and (aromatic) aldehydes in ethanol was prepared and stirred for 5 min. Then, catalyst (nano-Ni@zeolite-Y) was incorporated in the preparation [110]. *In pathway 5*, Shahbazi et al. explored a new sequence of bis-thiazolidinones. In this pathway, a solution of aldehydes, thioglycolic acid, ethylenediamine, and CuCr_2_O_4_ nanoparticles was refluxed in PhMe [111]. *In pathway 6*, Pal et al. arranged a new sequence of thiazolidinone analogues (4-oxo-2-(phenylimino) thiazolidin-5-ylideneacetate). In ethanol, a mixture of aniline, acetylenic ester, phenyl isothiocyanate, and nano-CuFe_2_O_4_ was agitated at room temperature [112]. *In pathway 7*, Sadeghzadeh et al. developed a new sequence of 1, 3-thiazolidin-4-one analogues. In this path, a solution of aldehydes, FeNi_3_-ILs-MNPs, amine, and thioglycolic acid was agitated at 50 °C [113]. *In pathway 8*, Safaei-ghomi et al. identified bis-thiazolidinones. A solution of aldehydes, nano-CdZr_4_(PO_4_)_6_, thioglycolic acid, and ethylenediamine was refluxed in toluene [114]. *In pathway 9*, Safaei-ghomi et al. prepared a new sequence of thiazolidinones. In this path, a solution of aldehydes, 2-mercaptoacetic acid, aniline, and nano-CdZr_4_-(PO_4_)_6_catalyst reacted under ultrasound radiations [115]. *In pathway 10*, Harale et al. arranged a novel sequence of 2, 3-disubstituted-4-thiazolidinones. In this pathway, a blend of (aromatic) aldehydes, aromatic amines, and mercaptoacetic acid was stirred at 100 °C with the addition of a catalytic amount of Pd NPs (palladium nanoparticles) [104].

## Anticancer activity profile of thiazolidin-4-one derivatives

More than 90% of deaths due to cancer occur by metastasis. For understanding the pathogenesis of cancer, apoptotic ways and directing cell proliferation are the standard methodologies [116–118]. The biological route involves the conversion of normal cells into cancerous cells which is a major approach used by researchers [119, 120]. During the treatment, drug resistance is the main obstacle, as resistance is developed against most of the chemotherapeutic mediators by means of diverse mechanisms like increase in drug export, restoration of drug-induced DNA damage, promotion of apoptosis, alteration of drug targets, and signaling transduction molecules [121]. Hence, there is an imperative necessity to grow new chemotherapeutic mediator to control the cancer because of augmented drug resistance.

**Fig. 8:**
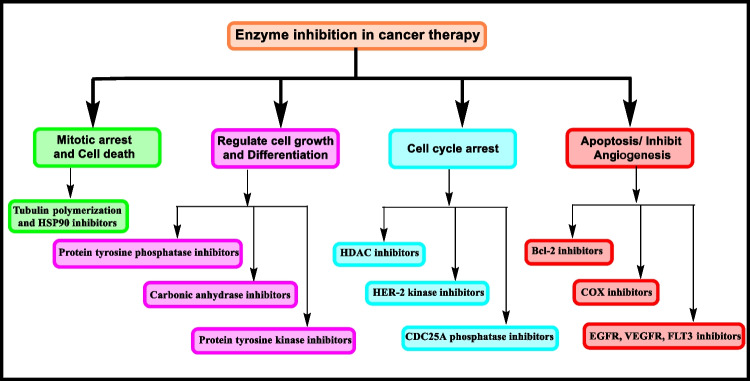
Possible role of enzyme inhinitors in cancer therapy

The possible mechanism of anticancer activity displayed by thiazolidinones involves inhibition of particular enzymes (Fig. 8). Among these, microtubules are polymers of tubulin that give structure and shape to eukaryotic cells. Therefore, inhibition of tubulin polymerization leads to mitotic arrest and cell death to prevent cancer progression. Carbonic anhydrases, protein tyrosine kinases, and protein tyrosine phosphatases are the enzymes responsible for various kinds of cancers. Inhibition of these enzymes regulates growth of cell and differentiation. Histone deacetylase inhibitors are the agents which lead to cell cycle arrest by regulating and altering gene expression. VEGFR are the receptors involved in angiogenesis and movement of monocytes and macrophages. EGFR and VEGFR are the members of tyrosine kinase family. Inhibition of EGFR and VEGFR regulates tumor progression by inducing apoptosis and by inhibiting angiogenesis. COX and BCl-2 are the proteins whose inhibition induces apoptosis.

### Anticancer activity by enzyme inhibition

Enzyme inhibition was recognized as a substitute and important objective for the management of tumors. Compounds containing thiazolidin-4-one were found effective in inhibition of a large number of enzymes or various enzymatic routes [7]. Protein/tyrosine kinases, (c-Met, CDK2, PIM kinases, AKt, Src, Ron, KDR, c-Kit and IGF-IR), carbonic anhydrases, HDAC, EGFR, HER-2, VEGFR2, CDC25A, BCl-2 protein, tubulin polymerization, and HSP90 are inhibited by thiazolidinone.

**Fig. 9:**
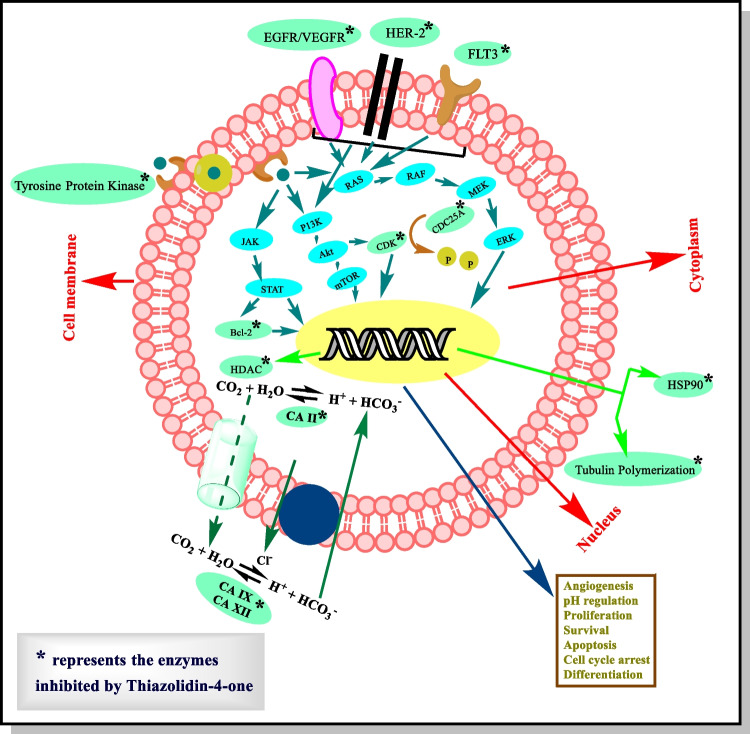
Various enzymatic pathways involved in cancer progression [131–138]

Histone deacetylases (HDACs) are identified as an important target for cancer management and are inhibited by histone deacetylase inhibitors (HDACi) [122]. HDACi are innovative chemotherapeutic agents that increase acetylation of histones by regulation of gene expression, alteration of gene expression, and also inducement of chromatin relaxation [123]. HDAC has revolutionized the production of a new class of pharmacological agents, HDAC inhibitors (HDACi), which are cytostatic agents that inhibit tumor cell proliferation in culture and persuaded cell cycle arrest, differentiation, and apoptosis in vivo [124]. Studies indicated that HDACi regulate cell proliferation and cell cycle progress by convincing growth arrest, apoptosis in tumor cells, and differentiation [123]. Histone tail’s lysine side chains were acetylated by histone acetyltransferases enzyme. In 2006, the first HDACi was “Zolinza” (SAHA, vorinostat) that acquired FDA (Food and Drug Administration) approval and is normally used in the management of T-cell lymphoma [125]. Several groups of HDACi were reported including benzamides, cyclic tetrapeptides, fatty acids (short-chain), benzofuranone, hydroxamic acid, boronic acid-based analogues, and sulfonamides comprising compounds and electrophilic ketones. Among these classes, HDACi based on hydroxamic acid was one of the foremost groups to which several studies have been described and it remains a dynamic research area [126]. Thus, HDACi has been depicted as an innovative class of chemotherapeutic drugs that control the expression of genes [124]. Angiogenesis is facilitated by vascular endothelial growth factor (VEGF) and its receptors. In a variety of cancers like renal cell, glioma, hepatocellular, and breast, VEGF is overexpressed [127]. Human protein kinases are the enzymes involved in cancer progression; therefore, protein kinases are the main objectives in the management of tumors [128]. Carbonic anhydrases catalyse the interconversion of H_2_O and CO_2_ into bicarbonate; this process is essential in mammals in various biological processes such as transporting carbon dioxide and maintaining the acid–base balance. Generally, carbonic anhydrases are overexpressed in various forms of cancer [129]. Protein tyrosine kinases are involved in various cellular functions like proliferation and differentiation both in normal cells and cancer cells. Studies revealed that ATP binding protein kinases are the attractive targets for the development of new chemotherapeutic agents [130]. The following text involves the description of various enzymes involved in cancer progression and the role of thiazolidin-4-one derivatives to inhibit these enzymes by interacting with them and their receptors. The various enzymes involved in cancer therapy are presented in Fig. 9 and discussed briefly in the following section:

#### Protein tyrosine kinase inhibitors

In normal and neoplastic cells, a large number of mitotic signaling ways were identified, in which protein kinases also present and play a crucial part in cell proliferation, cell growth, and metastasis and also induce anti-apoptotic effects in cancer cells. Stimulation of protein kinases was a common mechanism of cancer beginning. Tyrosine kinases are the enzymes which play a role in the transfer of phosphate group to the target proteins from adenosine triphosphate. There are two types of protein kinases-receptor protein kinases and non-receptor protein kinases. The receptor tyrosine kinases play a key role in the transfer of extracellular signals to the cytoplasm of cells. The blockage of the signaling pathways involved in cancer progression was a major approach for the development of drugs used as protein kinase inhibitors [139, 140].

**Fig. 10:**
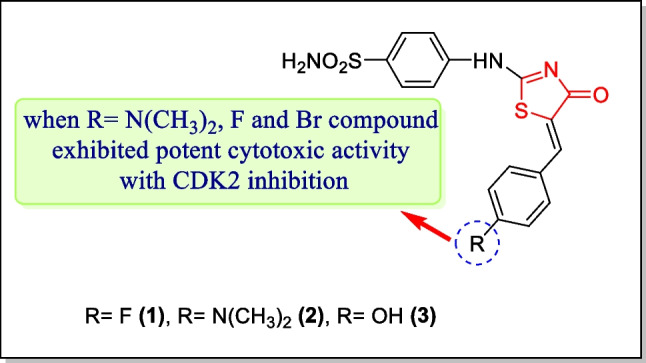
Thiazolidin-4-one analogues as CDK2 inhibitors with maximum cytotoxicity

In 2020, Nissan et al. have described an innovative sequence of various compounds in which compounds containing the 4-thiazolidinone nucleus also present. Anticancer action of such compounds was judged in response toMCF-7 (breast cancer cell line), HepG2 (liver cancer cell line), HCT-116 (colo-rectal cancer cell line), and A549 (lung cancer cell lines). Cytotoxic activity of all the prepared analogues was compared toward doxorubicin and 5-fluorouracil. Among the most potent analogues, compound **2** displayed CDK2 inhibitory activity (IC_50_ = 56.97 ± 2 µM) as well as potent cytotoxic activity in response to MCF-7 and HepG2 tumor cell lines, possessing IC_50_ value of 0.54 µM and 0.24 µM, respectively. Compounds **1** and** 2** displayed potent activity in response to MCF-7 with (IC_50_0.37 and 0.54 µM). Compounds **1**, **2**, and **3** (Fig. 10) exhibited good cytotoxic action towards the HepG2 cancer cell line having an IC_50_ value of 1.58, 0.24, and 2.28 µM, respectively. As analogue **2** displayed the most potent activity, it could be used by researchers in the future for the progression of promising anticancer mediators [141].

**Fig. 11:**
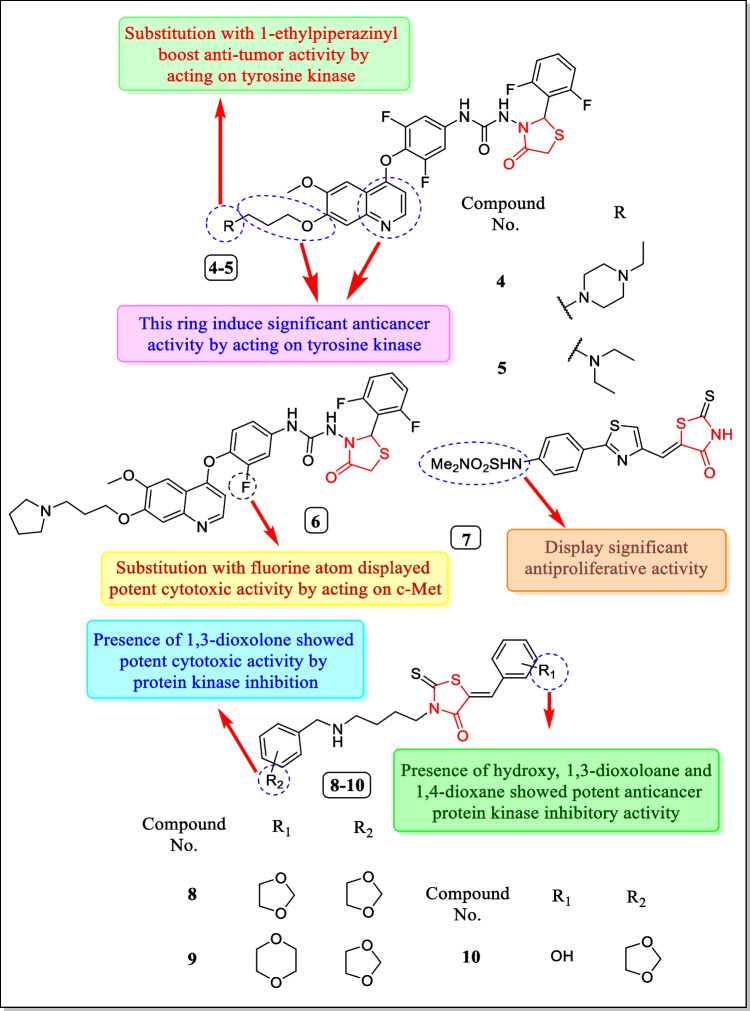
Novel thiazolidin-4-one analogues as tyrosine/protein kinase inhibitors

In the year 2018, Qi et al. studied a sequence of thiazolidin-4-one analogues and evaluated for tyrosine kinase inhibitory activity. Results concluded that analogue **4** was the most potent compound that showed multi-tyrosine inhibition in response to c-met kinase, Ron, c-Kit, KDR, c-Src, HER-2, IGF-1R, ALK, EGFR, and AXL with IC_50_ values of 0.015, 0.0029, 0.064, 0.85, > 10, > 10, 0.24, 2.1, 0.52, and 0.053 µM, respectively. The authors also estimated in vitro antitumor activity of the compound towards HT-29 (colon tumor cell line), A549 (lung tumor cell line), and MDA-MB-231 (breast carcinoma cell line) tumor cell lines. Results revealed that compound **4** reduced cell proliferation and induced apoptosis (IC_50_ = 0.073, 0.35, and 3.10 µM). Authors also investigated that the growth of two drug-resistant cell lines, A549^DDP^ (non-small cell lung carcinoma) and human breast adenocarcinoma (MCF^DR^), was inhibited by compounds **4** and **5** (Fig. 11) (IC_50_ = 0.35, 3.8 µM and 0.45, 3.4 µM) [142].

In 2018, Qi et al. in another study reported a novel sequence of N^1^-(2-aryl-1, 3-thiazolidin-4-one)-N^3^-aryl ureas and assessed for multi-tyrosine kinase inhibition activity. Among them, compound **6** (Fig. 11) acquires potent inhibitory activity toward multi-tyrosine kinases comprising c-Met, Src, Ron, KDR, c-Kit, and IGF-IR, etc., possessing IC_50_ = 0.021 µmol L^−1^. Compound **6** was assessed for cytotoxic activity towards (lung carcinoma cell line) A549 at 0.041 µM concentration. Molecular modeling study revealed that compound **6** binds completely with binding sites of c-Met kinase enzyme and formed one weak hydrogen bond and two strong hydrogen bonds with ATP binding site [143].

In 2017, a new sequence of thiazolidin-4-one analogues was investigated and assayed by Bataille et al. for their PIM kinase inhibition. The synthesized sequence was found to be excellent PIM pan-inhibitors. Compound **7** (Fig. 11) showed potent PIM kinase inhibition (IC_50_ = 2.2 ± 1.8 µM). Compound **7** also displayed anti-proliferative activity with regard to two leukemia cell lines (MV4-11 and K562) having IC_50_ values of 3.4 µM and 0.75 µM [144].

In 2015, a novel series of 3-(4-arylmethyl-amino)butyl-5-arylidene-2-thioxo-1,3-thiazolidin-4-ones were arranged by Dago et al. and estimated for protein kinase inhibitory activity. Out of the tested analogues, compounds **8**, **9**, and **10** (Fig. 11) possessed potent protein kinase inhibitory activity like HsCDK5/p25, SsGSK-3α/β, SsCK1δ/ε, and HsHaspin having IC_50_ values of 1.1 µM, > 10 µM, 6.6 µM, and > 10 µM, respectively, for compound **8,** 1.3 µM, > 10 µM, 5.1 µM, and > 10 µM, respectively, for compound** 9,** and > 10 µM, > 10 µM, 1.4 µM, and > 10 µM, respectively, for compound **10**. The prepared analogues were assayed for anticancer action in response to seven carcinoma cell lines including Huh7 D12 (hepatocellular carcinoma cell line), MDA-MB-231 (breast cancer cell line), Caco 2 (colo-rectal adenocarcinoma cell line), PC3 (prostate cancer cell line), HCT 116 (colo-rectal cancer cell line), HaCat (keratinocyte cell line), NCI-H727 (lung cancer cell line), and fibroblast [145].

**Fig. 12:**
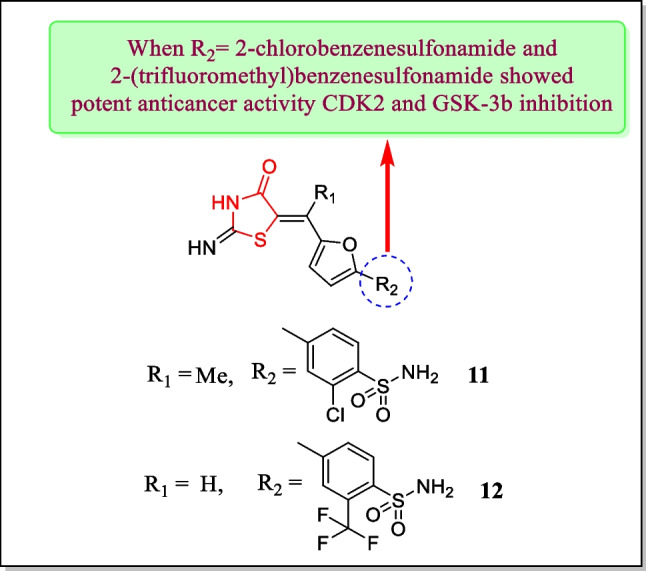
Thiazolidin-4-one containing analogues as potent CDK2 and GSK-3β inhibitors

In 2007, Richardson et al. explored a newer series of thiazolidin-4-one comprising CDK2 inhibitors and assayed for CDK2 and GSK-3β inhibitory activity. Compound **11** (Fig. 12) was found to express CDK2 (IC_50_ = 0.03 µM), and compound **12** (Fig. 12) showed GSK-3β inhibitory activity (IC_50_ = 0.002 µM). Molecular docking study was performed in two stages using rDock and rCat to bind the compounds against CDK ATP binding site. The molecular modeling study concluded that compounds form H-bond with the protein [146].

#### Carbonic anhydrase inhibitors

In prokaryotes and eukaryotes, carbonic anhydrases are the abundant zinc enzymes having four distant gene families: αCAs, βCAs, γCAs, and δCAs. CA IX was the first carbonic anhydrase reported to be linked with cancer. The second carbonic anhydrase co-expressed with CA IX in various tumor cells is CA XII. In cancer therapy, targeting cancer-linked enzymes is an encouraging strategy [147].

**Fig. 13:**
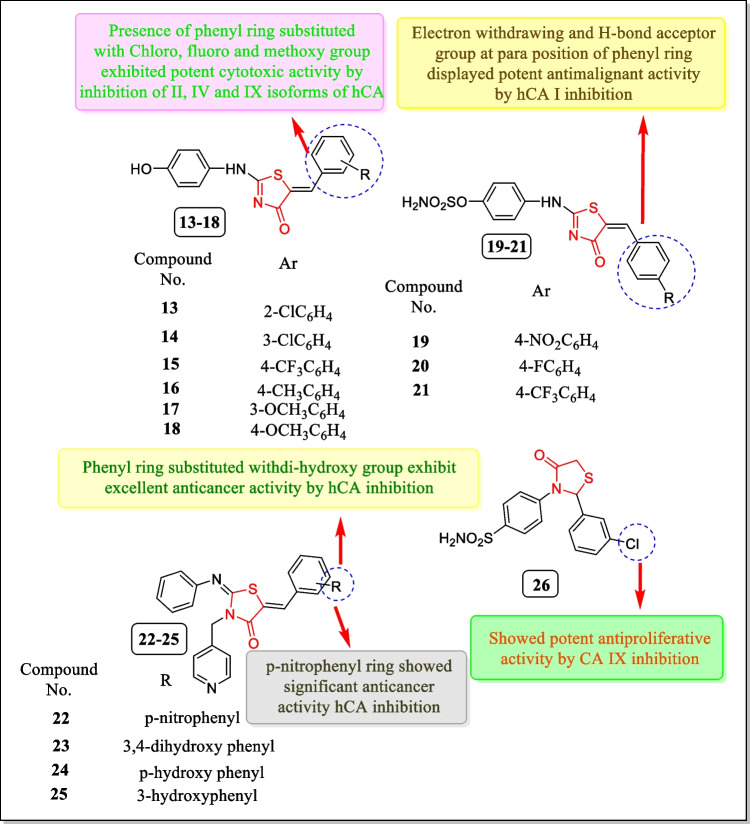
Thiazolidin-4-one analogues as carbonic anhydrase inhibitors with a targeted response

In 2018, Nocentini et al. presented thiazolin-4-one based aromatic sulfamates series and assayed for carbonic anhydrase inhibitory activity (hCA I, II, IV, and IX). Results indicated that compounds **16** and **18** strongly inhibited hCA II isoform of carbonic anhydrase (*K*_i_ 76 and 90 µM respectively) and compounds **14** and **15** inhibited hCA IX isoform only (*K*_i_ 30 and 22 µM respectively), whereas compounds **13** and **17** inhibited hCA IV isoform of the enzyme with *K*_i_ 43 and 39 µM respectively and compounds **19**, **20**, and **21** displayed potent inhibitory activity against hCA I having *K*_i_s values of 388.6, 311.6, and 158.1 nM, respectively [148]. The structures of analogues **13–21** are demonstrated in Fig. 13.

In 2017, Ansari et al. introduced an innovative class of pyridine-thiazolidinone analogues as powerful carbonic anhydrase IX inhibitors. Results indicated that among all tested analogues, compounds **22** and **23** inhibited the activity of carbonic anhydrase IX enzyme. The prepared analogues were also tested for cytotoxic activity in response to breast cancer cell line MCF-7 and liver cancer cell line HepG-2. Out of the most potent compounds, compounds **22**, **23**, and **24** were found to show powerful inhibitory activity toward MCF-7 cell line (IC_50_ = 18.9 ± 2.19 µM, 13.0 ± 2.28 µM, and 12.4 ± 1.39 µM) and compounds **22**, **23**, **24**, and **25** showed superior activity against HepG-2 cell line (IC_50_ = 11.8 ± 1.95 µM, 18.9 ± 1.34 µM, 16.2 ± 1.34 µM, and 17.6 ± 2.12 µM). A molecular modelling study of the compounds **22** and **23** revealed that in compound **22,** nitrobenzylidene ring created 6 hydrogen bonds with amino acid residues of enzymes like His94, His96, Thr199, and Thr200 while the thiazolidin-4-one ring created one hydrogen bond with active site residue Thr200 of enzyme. Similarly, with the dynamic site residues, compound **23** created seven hydrogen bond interactions and hydrophobic interactions [149] (Fig. 13).

In 2013, a new series of 4-(4-oxo-2-arylthiazolidin-3-yl) benzenesulfonamide analogues were explored by Suthar et al. and assayed for their CA IX inhibitory activity. Among them, compound **26** (Fig. 13) displayed potent CA IX inhibition (*K*_i_ = 2.2 nM). Results concluded that compound **26** also displayed anti-proliferative activity in response to (colon adenocarcinoma cell line) COLO-205, (breast cancer cell line) MDA-MB-231, and (prostate cancer cell line) DU-125 (IC_50_ = 5.03 µg/mL, 5.81 µg/mL, and 23.93 µg/mL, respectively). Molecular modeling study demonstrated that compound **26** strongly interacts with the active site of the CA IX. Sulfonamide pharmacophore interacted with amino acid residues or residues of targeted proteins, e.g., Asn-62, His-64, Gln-67, Gln-92, and Thr-199 [150].

#### CDC25A phosphatase inhibitors

CDC25 (cell division cycle phosphatase) is a member of protein tyrosine phosphatases involved in the activation of cyclin-dependent kinases. For cell proliferation, proper regulation and activity of CDC25 are essential. Generally, three analogues of CDC25 are present in humans which are CDC25A, CDC25B, and CDC25C. As CDC25 involves tumor growth, consequently, it is an interesting target for expansion of anticancer drugs [151].

**Fig. 14:**
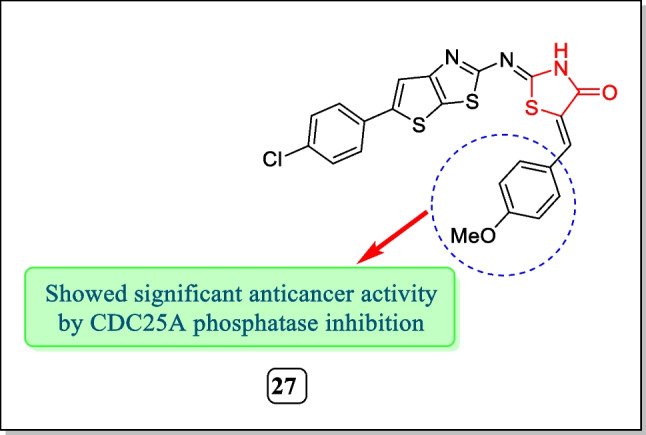
Anti-proliferative potential of thiazolidin-4-one analogues as CDC25A inhibitors

In the year 2016, Huber-Villaume et al. synthesized a sequence of 2-(thienothiazolylimino)-1, 3-thiazolidin-4-one analogues and assayed for their cell division cycle phosphatase (CDC25) enzyme inhibitory activity. Results concluded that compound **27** (Fig. 14) showed potent CDC25A inhibitory activity having an IC_50_ value of 6.2 ± 1.0 µM [152].

#### HDAC inhibitors

Histone deacetylase (HDAC) is an essential enzyme for removing the acetyl group from residues of lysine to the ends of histone proteins and generates positive charge which ultimately triggers the DNA’s negatively charged phosphate backbone to strongly coil and control chromatin arrangements [153]. HDACi (histone deacetylase inhibitors) are found to be active curative mediators that kill melanoma cells by preventing the functions of histone deacetylase (HDAC) or by varying chromatin arrangements [126]. HDACs bind with DNA via multi-protein complexes but not directly. HDAC inhibitors are compounds that induce cell growth arrest and cell death, and therefore are widely used as anticancer agents. HDAC inhibitors generally involve three common structural components, i.e., zinc-binding moiety, a straight-chain alkyl, aryl or vinyl linker, an opposite capping group. A large range of HDAC inhibitors was used in combination with other anticancer agents for the management of malignancy [154]. Recent discoveries show that HDACi specifically induces differentiation, apoptosis, and growth arrest in melanoma cells through transcriptional stimulation of genes (a small set) that regulate cell proliferation as well as the production of cell cycles such as p21 [123].

**Fig. 15:**
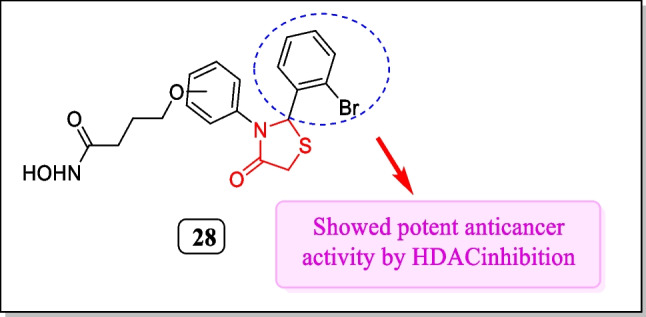
Thiazolidin-4-one analogues as HDAC inhibitors showed effective inhibition

In 2016, Yang et al. designed a sequence of thiazolidinones and verified for their histone deacetylase (HDAC) inhibitory activity. Major findings indicated that only compound **28** (Fig. 15) displayed HDACi with an IC_50_ value of 73 ± 14. Vorinostat was employed as a reference and the outcomes specified that HDAC1 inhibition depends on linker-length. Synthesized compounds also assayed towards four tumor cell lines, HeLa (cervical carcinoma cell line), MCF-7 (breast cancer cell line), LNCaP (prostate carcinoma cell line), and A549 (lung cancer cell line). Compound **28** showed the most powerful response to all cancer cell lines (IC_50_ = 3.2 ± 0.5, 2.1 ± 0.5, 2.9 ± 0.3, and 4.6 ± 0.8 µM, respectively). Results concluded that compound **28** not only showed anti-proliferative activity and impeded colony formation of tumor cell lines but also appeared to be superior inhibitors of metastasis of cancer cell lines [155].

#### Bcl-2 inhibitors

Apoptosis is a main type of PCD (programmed cell death). PCD is essential for mature organisms to sustain cell homeostasis. Bcl-2, a component of protein, is essential for the control of apoptosis. The elevated level of Bcl-2 proteins is liable for the cancer induction in cells; therefore, inhibition of Bcl-2 is vital to suppress cancer.

**Fig. 16:**
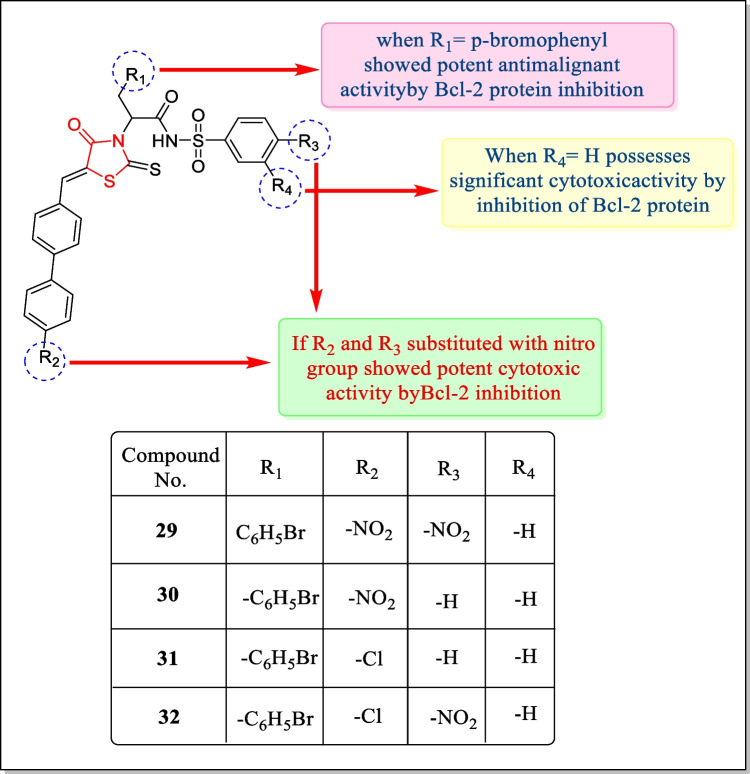
Thiazolidin-4-one analogues with inhibitory effect against Bcl-2 inhibitors

In the year 2015, Wan et al. arranged a new sequence of new 2-thioxo-4-thiazolidinone analogues and screened for their cytotoxic potential as Bcl-2 inhibitors. Among compounds, compound **29** inhibited Bcl-2 protein as well as myeloid cell leukemia sequence1 (Mcl-1) protein possessing a *K*_i_ value of 0.36 ± 0.03 µM. Also, compounds **29**, **30**, **31**, and **32** are estimated for anticancer activity towards three human tumor cell lines including breast tumor cell line MDA-MB-231, prostate tumor cell line Pc-3, and leukemia cell line K562 (IC_50_ = 30.38 ± 2.9, 40.67 ± 0.04, 7.90 ± 1.70 µM, respectively, for compound **29;** and 46.15 ± 2.94, > 50, > 50 µM, respectively, for compound **30;** and 28.77 ± 1.01, > 50, 20.89 ± 1.48 µM, respectively, for compound **31;** and 28.09 ± 4.39, 24.09 ± 2.75, 9.44 ± 2.34 µM, respectively, for compound **32,** respectively). Results concluded that all the compounds **29**–**32** showed better anti-proliferative action contrary to all cancer cell lines [156]. Graphical representation of the structures is reflected in Fig. 16.

#### Tubulin polymerization and HSP90 inhibitors

Microtubules are the proteins and components of mitotic spindles having a pipe-like structure containing α, β-heterodimers involved in cell division. Microtubules are made up of two subunits of globular proteins, i.e., α and β-tubulin (molecular weight 50 kDa). A large number of microtubule-associated proteins like MAP2, MAP4, STOP, tau, and Mip-90, each having a mass of 20 kDa are involved in formation and stability of microtubules [157]. Microtubules involve cell growth, and cell proliferation, invasion, and migration. Microtubule-inhibiting agents are the major agents used to develop chemotherapeutic agents to combat the above cellular processes. There are two categories of microtubule-inhibiting agents: agents that inhibit the polymerization of microtubules and agents that promote or stabilize microtubules [158]. Tubulin protein has continuously been a significant, critical, and practical goal for the expansion of cytotoxic drugs [159].

**Fig. 17:**
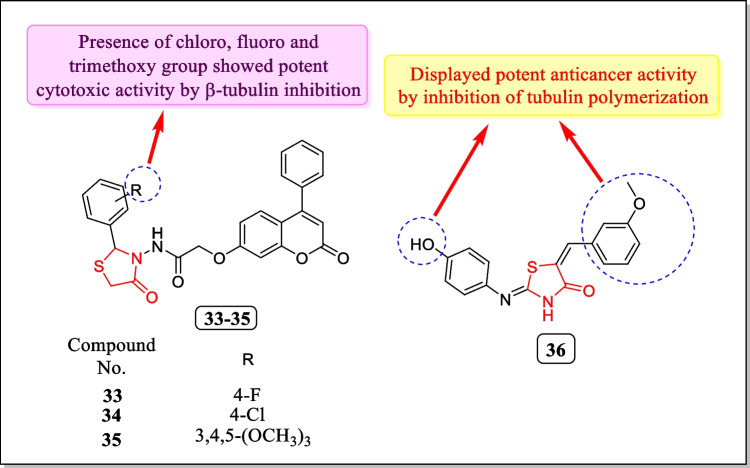
Thiazolidin-4-one as tubulin polymerization inhibitors showed potent anticancer effect

In 2018, a novel series of 4-phenylcoumarin derivatives containing thiazolidinone nucleus was designed by Batran et al. Each prepared analogue has been assayed for tubulin polymerization inhibition action. Thiazolidin-4-one containing derivatives **33**, **34**, and **35** found strong tubulin polymerization inhibitor (IC_50_ = 9.37, 2.89, and 6.13 μM). Results indicated that compounds **33**, **34**, and **35** (Fig. 17) displayed strong cytotoxic activity in response to breast cancer cell line MCF-7 (IC_50_ value of 11.1, 16.7, and 21.2 µg/mL). The molecular modeling study discovered that compounds **33**, **34**, and **35** exhibited binding with pockets of the crystal structure of β-tubulin (a protein present in mitotic spindles) as showed by colchicine [160].

In 2015, Mu et al. reported the new sequence of tubulin polymerization inhibitor MHPT displayed discriminating anti-proliferative action in response to rhabdomyosarcoma in vitro and in vivo and assessed for anti-RMS action by high-throughput cytotoxicity screening. A new synthetic analogue **36** (Fig. 17) (MHPT) was found as a powerful and discriminating anti-rhabdomyosarcoma mediator. About 50% of the progression of the rhabdomyosarcoma tumor cell lines, RD and SJ-RH30, was inhibited by MHPT, at 0.44 ± 0.19 μM and 1.35 ± 0.58 μM, respectively. The above findings indicated that because of the low toxicity level of MHPT, it has the prospective to be auxiliary established into a discriminating anti-rhabdomyosarcoma medication [161].

**Fig. 18:**
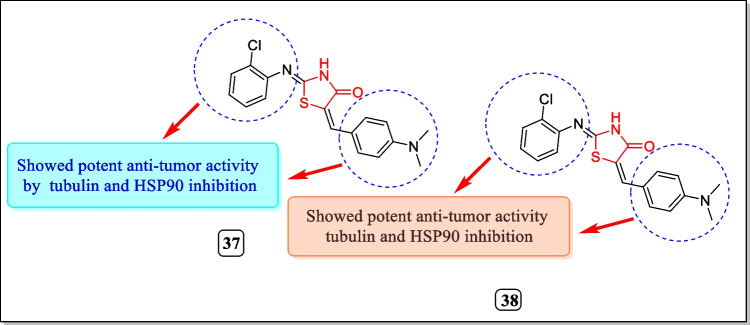
Thiazolidin-4-one analogues as tubulin and HSP90 inhibitors

In 2014, Zhang et al. produced a CDBT (2-(2-chlorophenylimino)-5-(4-dimethylaminobenzylidene) thiazolidin-4-one) compound (**37)** (Fig. 18) and checked for its tubulin and HSP90 (heat shock protein 90) dual inhibitory activity using non-small cell lung tumor model. Results identified that the compound was identified as an excellent inhibitor of both tubulin and HSP20. CDBT compound **37** also evaluated against P-gp, paclitaxel resistant, and paclitaxel susceptible (lung cancer cell line) NSCLC H460_TaxR_ and H460 cells (IC_50_ 0.8 mM and 0.7 mM). HSP90 proteins like CRAF-1 and ERBB2 and α and β-tubulin proteins were also corrupted by CDBT compound **37**. Results concluded that the activity of H460_TaxR_ is lesser than that of H460 cells. CDBT treated with Na_3_VO_4_ express no P-gp ATPase activity but in nonappearance of Na_3_VO_4_ showed better P-gp ATPase activity [162].

In another study (2013), Zhang et al. again synthesized CDBT compound **38** (Fig. 18) and assayed for their dual tubulin and HSP90 inhibitory activity. The resulted data suggested that both α, β-tubulin proteins and HSP90 proteins such as CRAF-1 and ERBB2 in NSCLC cells (H460 with IC_50_ = 0.7 µM and H322 having IC_50_ = 1.0 µM). Both the cells incubated with CDBT for 14 h showed corrupted filaments and small microtubule fragments. The molecular modeling study suggested that CDBT binds to the colchicine site and restricted its binding to colchicine pockets [163].

#### EGFR, VEGFR, FLT3, and HER-2 kinase inhibitors

VEGF kinase receptors present in human beings comprise VEGFR-1, VEGFR-2, and VEGFR-3. Additionally, VEGFR belongs to the family of tyrosine kinases. VEGFR-1 is involved in the movement of monocytes and macrophages, while VEGFR-3 involves lymph angiogenesis [164]. Angiogenesis is a complex and highly controlled process crucial for tumor growth and metastasis [165]. The EGFR (epidermal growth factor receptor) which is a constituent of the ErbB family also comprises the HER-1, EGFR-2/HER-2, HER-3, and HER-4 tyrosine kinase receptors. Generally, VEGFR-2 is essential for the development of both local and distant cancer growth [166].

**Fig. 19:**
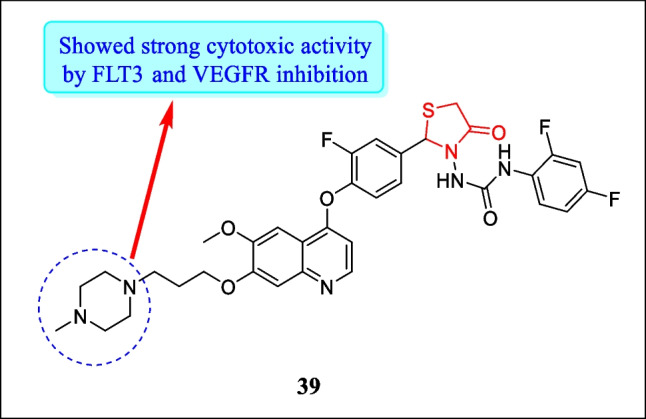
Anticancer potential of thiazolidin-4-one as FLT3 and VEGFR inhibitors

In year 2019, Qi et al. designed a sequence of innovative class of thaizoldin-4-one urea analogues and investigated for their FLT3 and VEGFR2 inhibitory action. Results proved that compound **39** (Fig. 19) acts as a multi-kinase inhibitor and presented powerful inhibitory activity against FLT3 and VEGFR2 having an IC_50_ 18.7 µM. From molecular docking, the way of binding of compound **39** with FLT3 kinase was investigated by molecular operating environment software to find out the structural resistance mechanism. Results of molecular docking revealed that with FLT3 compound **39** exhibited type II binding mode. In compound **39,** two (hydrogen) bonds have been formed by urea moiety with Asp698 residues. Furthermore, the strength of binding of the compound was raised by three arene-H-interactions, one among Leu616 and benzene ring (O-linked) and the two other interactions among Val624 and quinoline ring. All the analogues were assayed for cytotoxic action in response to A549 (lung cancer cell line) and HT-29 (colon tumor cell line). The most active derivative **39,** which was a FLT3/VEGFR2 dual inhibitor, showed strong anticancer activity (by suppressing cell growth) toward A549 and HT-29 tumor cell lines with an IC_50_ value of 0.65 µM and 0.11 µM, respectively. Results also exhibited that cytotoxic activity of compound **39** was stronger and long-lasting as compared to cabozantinib and compound **39** also inhibited cell migration more efficiently in contrast to cabozantinib after 48 h of treatment [167].

**Fig. 20:**
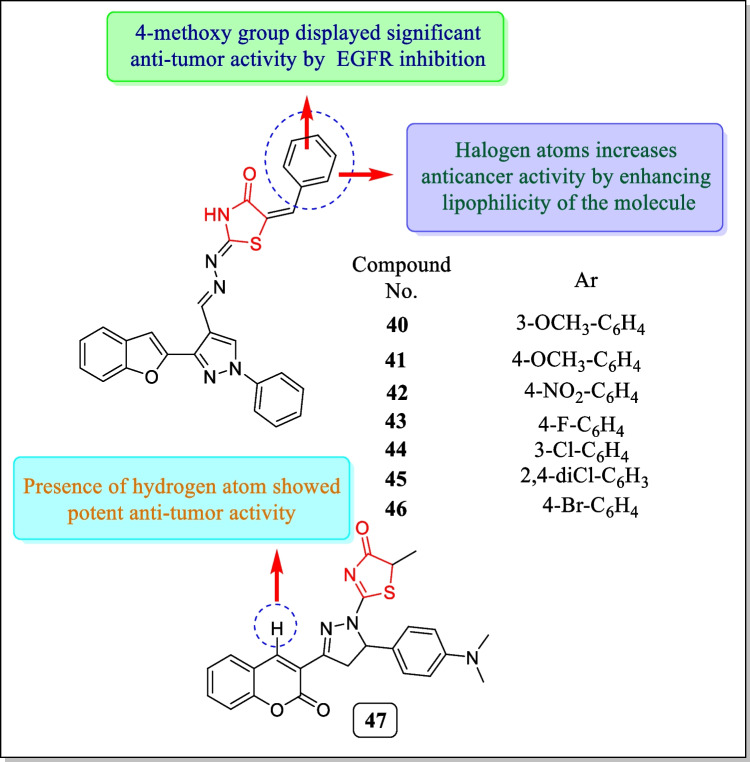
Thiazolidin-4-one comprising analogues as inhibitors of EGFR and VEGFR2

In 2019, Abbas et al. introduced a newer series of benzofuran–pyrazole-hydrazono- thiazolidin-4-one hybrids and tested for EGFR inhibitory performance. The consequences concluded that compound **42** showed strong EGFR inhibition (IC_50_ = 0.07 µM) and also exhibited arrest of the cell cycle in the G1/S phase in (cervical cancer cell line) HeLa. The molecular modeling study showed that compound **42** interacts with vital amino acids of EGFR enzyme active sites to display potent inhibition of the enzyme. Among all the screened compounds, compounds **40**–**46** (Fig. 20) exerted active anticancer action towards the HeLa cancer cell line having IC_50_ value between 0.60 and 2.99 µM [168].

In 2019, VEGFR2 kinase inhibitory activity of a new sequence of thiazolylpyrazolyl coumarin analogues was assayed by Mohamed et al. In the synthesis, compounds containing thiazolidin-4-one nucleus also present. Thiazolidin-4-one containing compound **47** (Fig. 20) exhibited potent VEGFR2 kinase inhibitory activity (IC_50_ = 0.1696 ± 0.0104 µM). Compound **47** also possessed strong anti-proliferative action in response to MCF-7 possessing IC_50_ value of 10.75(4.80 ± 0.47) µg/mL. The molecular modeling results revealed that analogue **47** effectively binds with the active site of the VEGFR2 kinase enzyme [169].

**Fig. 21:**
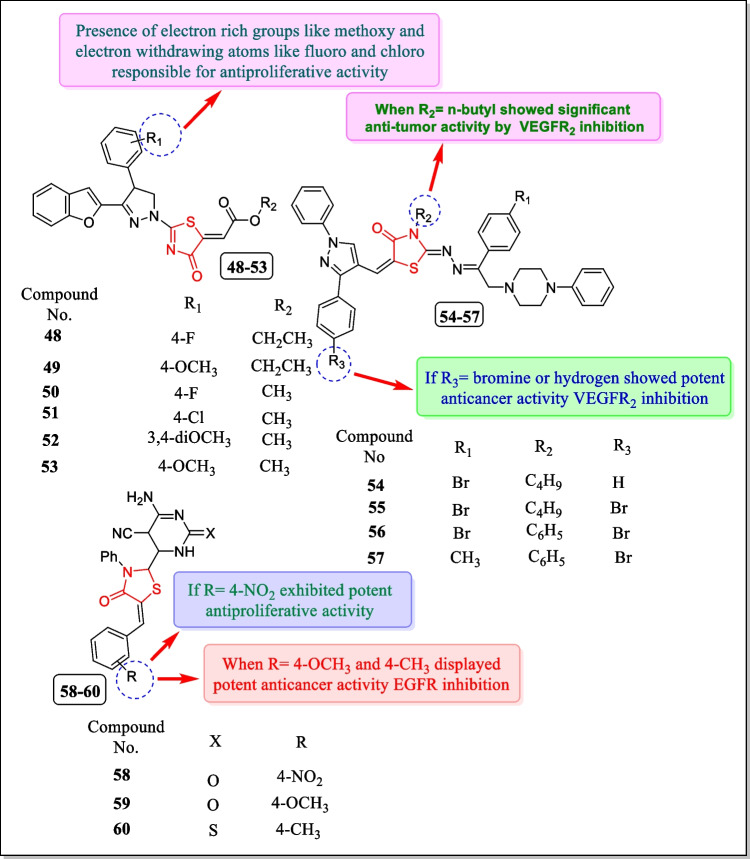
A newer sequence of 4-thiazolidinone analogues as EGFR and VEGFR inhibitors

In 2018, a newer series of pyrazolo oxothiazolidine analogues were reported by Yakaiah et al. and assayed for EGFR and VEGFR2 inhibitory action. Analogues **48**–**53** (Fig. 21) presented the most impressive EGFR and VEGFR2 inhibitory activity along with anticancer activity with regard to A549 human lung carcinoma cell line (IC_50_ 0.930 µg/mL, 1.207 µg/mL, 0.808 µg/mL, 1.078 µg/mL, 0.967 µg/mL, and 2.445 µg/mL, respectively). The molecular docking study discovered that all the potent analogues effectively bind to catalytic sites of EGFR and VEGFR2 enzymes. From in vitro results, compound **50** found potent EGFR and VEGFR2 inhibitor as it formed strong H-bond with MET 769 (hepatocyte growth factor receptor) [170].

In 2017, El-Miligy et al. described a novel sequence of piperazine-based thiazolidinone analogues and tested for VEGFR2 tyrosine kinase inhibitory action. Outcomes indicated that analogues **54**, **55**, **56**, and **57** (Fig. 21) were found as the most potent VEGFR2 tyrosine kinase inhibitors. For molecular modeling study of the most persuasive compounds in the VEGFR2 kinase domain, AUTODOCK software is used. For the docking study, the VEGFR2 kinase domain of *Homo sapiens* was acquired from the protein databank. Receptor for AUTODOCK software was prepared by adding all hydrogens and by deleting water molecules and ligands. The reported compounds were also assayed for cytotoxic activity in response to the HepG2 cell line. The most potent analogues **54**, **55**, **56**, and **57** exhibited good activity in response to the HepG2 cell line (IC_50_ = 0.03–0.06 µM) [171].

In 2013, the antiproliferative activity of a new sequence of triazaspiro [4, 5] dec-8-ene benzylidine derivatives comprising the thiazolidinone ring system was assayed for their EGFR inhibitory activity by Fleita et al. Among them, compound **58** displayed the greatest powerful EGFR inhibitory action with an IC_50_ value of 6.355 µM. Analogues **59** and **60** expressed strong cytotoxic activity in response to MCF-7 (IC_50_ = 10.8 ± 2.6 µM). The molecular modeling study demonstrated that compound **58** showed hydrogen binding interaction with ATP molecule [172]. The structures of the analogues **58–60** are displayed in Fig. 21.

**Fig. 22:**
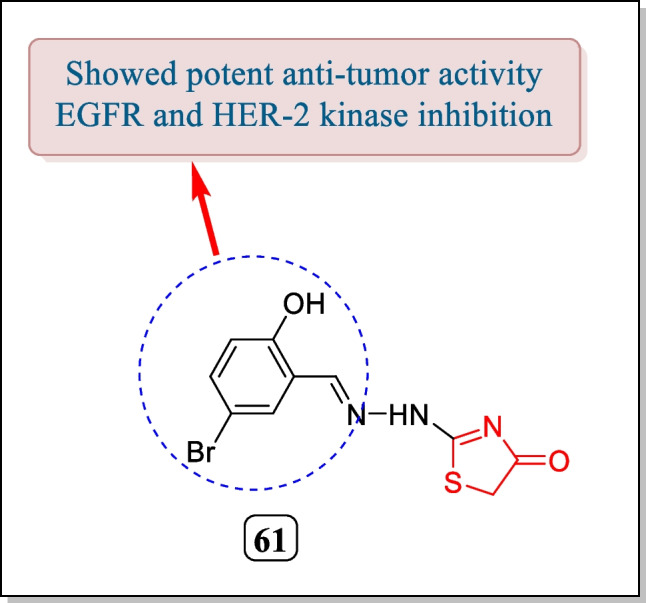
Derivatives having thiazolidin-4-one nucleus as EGFR and HER-2 inhibitor

In 2010, Lv et al. evaluated two sequences of thiazolidinones for EGFR and HER-2 kinase inhibition action. Only analogue **61** (Fig. 22) was found to exhibit strong EGFR and HER-2 kinase inhibition (IC_50_ = 0.09 µM and 0.42 µM, respectively). Analogue **61** also showed anti-proliferative action in response to breast carcinoma cell line MCF-2 in contrast to erlotinib used as the reference drug. The outcomes of molecular docking showed that compound **61** effectively anticipated with hydroxyl group against a mercapto group of Met-769 and also H-bond is formed by nitrogen atom of thiazolidin-4-one with mercapto group of Cys 751. Thus, compound **61** could be used by scientists to produce effective anticancer drugs for cancer treatment in upcoming years [173].

#### COX inhibitors

Cyclooxygenase (COX) is an enzyme that plays an important role as a catalyst for the transformation of arachidonic acid to prostaglandins (PGs) in the cell. PGs play a vital role in cancer cell progression. This enzyme is divided into two different isoforms, i.e., COX-1 and COX-2.

**Fig. 23:**
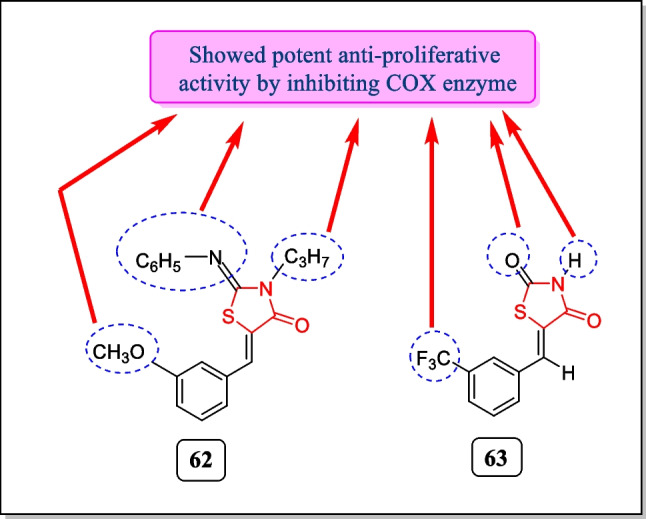
Thiazolidin-4-one analogues as COX-inhibitors with significant cytotoxic potential

In 2005, Ottana et al. correlated COX-2 inhibitory properties of some thiazolidin-4-one derivatives by performing in vitro anti-proliferative activity of these compounds. Among them, 2-phenylimino-5-(3-methoxyphenylmethylidene)-3-propyl-4-thiazolidinone **62** (Fig. 23) was found most effective with selective COX-2 inhibitory activity toward the colon carcinoma cell line HT29 (IC_50_ value 64.1 µM at 72 h and 23.4 µM at 96 h) and analogue **63** displayed potent cytotoxic activity toward all tumor cell lines (IC_50_ = 38.8 µM at 72 h and 59.7 µM at 96 h). Analogue **62** is also active against human colorectal adrenocarcinoma cell lines, DLD-1 and HCT-116, cell lines that do not explicit COX-2 having IC_50_ values 13.1 and 34.2 µM, respectively, and analogue **63** (Fig. 23) especially showed potent anti-proliferative activity against DLD-1 (IC_50_ = 17.8 µM at 96 h) [174].

#### Protein tyrosine phosphatase inhibitors

**Fig. 24:**
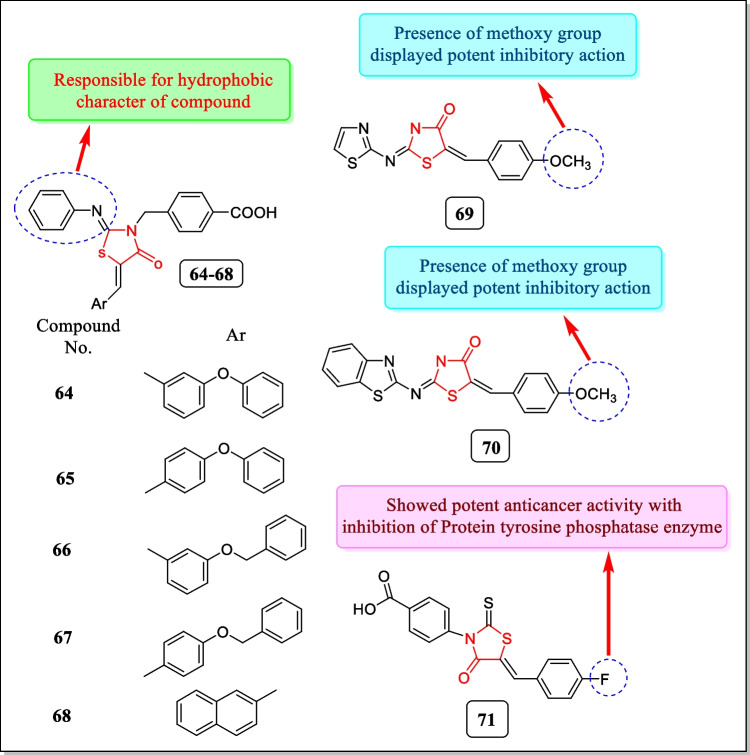
Thiazolidin-4-ones active against protein tyrosine phosphatase

In another study (2009), Ottana et al. prepared a novel series of 4-[(5-arylidene-4-oxo-2-phenyliminothiazolidin-3-yl) methyl]-benzoic acids for evaluation of their inhibitory activity against human protein tyrosine phosphatase (PTP1B) and low molecular weight PTP (LMW-PTP) enzymes. In some kinds of human cancers, for example, ovarian and breast tumors and epithelial carcinomas, PTP1B is overexpressed and thus can work as a positive controller of tumor beginning and development. In this study, good levels of this PTP have been found in colon tumors, breast, and neuroblastoma. Experiments involving molecular modeling studies of the compounds within the binding spots of both enzymes were carried out. Compounds **64** to **68** (Fig. 24) were found to have significant inhibitory performance toward both human PTP1B (IC_50_ = 1.9 ± 1.0, 1.1 ± 0.1, 3.8 ± 0.1, 1.1 ± 0.1, 1.9 ± 1.0 µM) and IF1 isoform of human LMW-PTP (IC_50_ = 5.4 ± 0.5, 3.1 ± 0.4, 3.1 ± 0.5, 2.7 ± 0.2, 3.7 ± 0.2 µM) in the low micro-molar range [175].

SHP-2 is a PTPN11 gene encoded non-receptor protein tyrosine phosphatase, and mediates cell signaling by growth factors and cytokinesis through the Ras-MAP (mitogen-activated protein) kinase pathway. In 2008, Geronikaki et al. synthesized 2-thiazolylimino/heteroarylimino-5-arylidene-4-thiazolidinone analogues. The newly synthesized analogues having thiazole, benzothiazole, and benzoisothizole rings were divided into three series. These compounds were assessed for HP-2 inhibitory activity by means of human recombinant GST-fusion SHP-2. Compound 4-methoxy-substituted thiazole derivative **69** (Fig. 24) (*K*_i_^a^ = 11.7 µm) and benzothiazolyl analogue **70** (Fig. 24) (*K*_i_^a^ = 11.7 µm) showed the most potent inhibitory activity [176].

Protein tyrosine phosphatase family (PTPs) regulates various cell mechanisms. Dual-specificity phosphatases (DSPs) are a subclass of PTP family and have received the attention of researchers for a possible role in cancer treatment. In 2005, Cutshall et al. prepared a series of rhodanine-based inhibitors. Newly synthesized analogues were assayed to identify inhibitors towards the DSP family member JNK-stimulating phosphatize-1 (JSP-1). From the study, compound **71** (Fig. 24) having 5-benzylidene-3-phenyl-2-thioxo-thiazolidin-4-one (rhodanine) core exhibited the most potent inhibitory action (IC_50_ = 1.3 µM) [177].

### Additional anticancer activities of thiazolidin-4-one derivatives

**Fig. 25:**
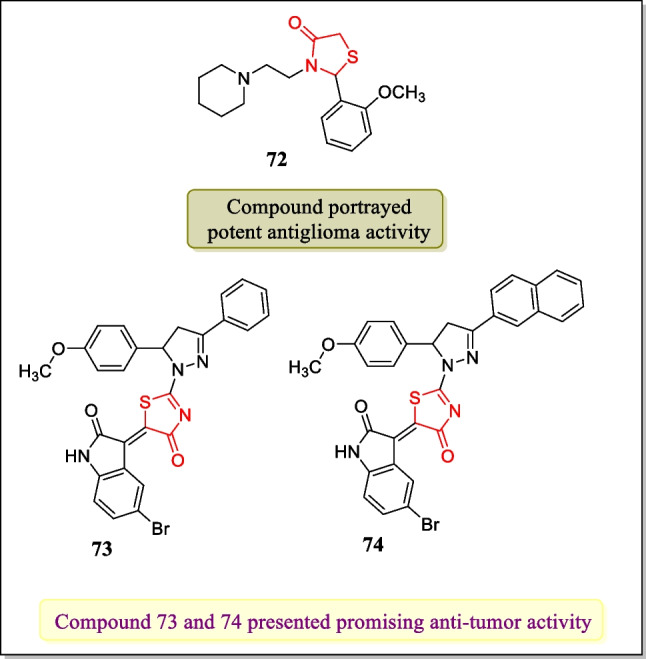
Thiazolidin-4-one loaded nanocarriers as effective cytotoxic agents

Current scientific literature evidences that thiazolidin-4-one analogues have demonstrated significant cytotoxic activity. A large number of scientists across the globe are pursuing their research endeavors to explore the maximum medicinal utility of these heterocycles as possible anticancer agents. This review paper is an intense endeavor to compile and present vital reports from recent scientific literature related to anticancer activity exhibited by thiazolidin-4-one heterocyclic compounds and their derivatives. Important reports from scientific literature related to the anticancer action of thiazolidin-4-one analogues are presented in the following text:

In 2019, Silveira et al. presented 2-(2-methoxyphenyl)-3-((piperidin-1-yl) ethyl) thiazolidin-4-one which have loaded by polymeric nanocapsules and investigated them for in vitro antiglioma activity and in vivo toxicity. Results indicated that treatment of human U138MG and rat C6 cell lines with analogue **72** (Fig. 25) (thiazolidin-4-one nanoparticles) reduced viability and proliferation of cell line more efficiently as compared to normal thiazolidin-4-one derivative at 6.25–50-μM concentration [178].

In 2018, Kobylinska et al. investigated new thiazolidin-4-one compounds complexed with PEG-containing polymeric nanocarrier. Analogues **73** and **77** (Fig. 25) (Les-3288, Les-3833) were estimated for their antineoplastic activity toward glioma C6 cells at 0.1 µg/mL, 0.5 µg/mL, and 1.0 µg/mL concentrations. Outcomes represented that analogues **73** and **74** (Les-3288, Les-3833) were found the most active with limited harmful effects [169].

**Fig. 26:**
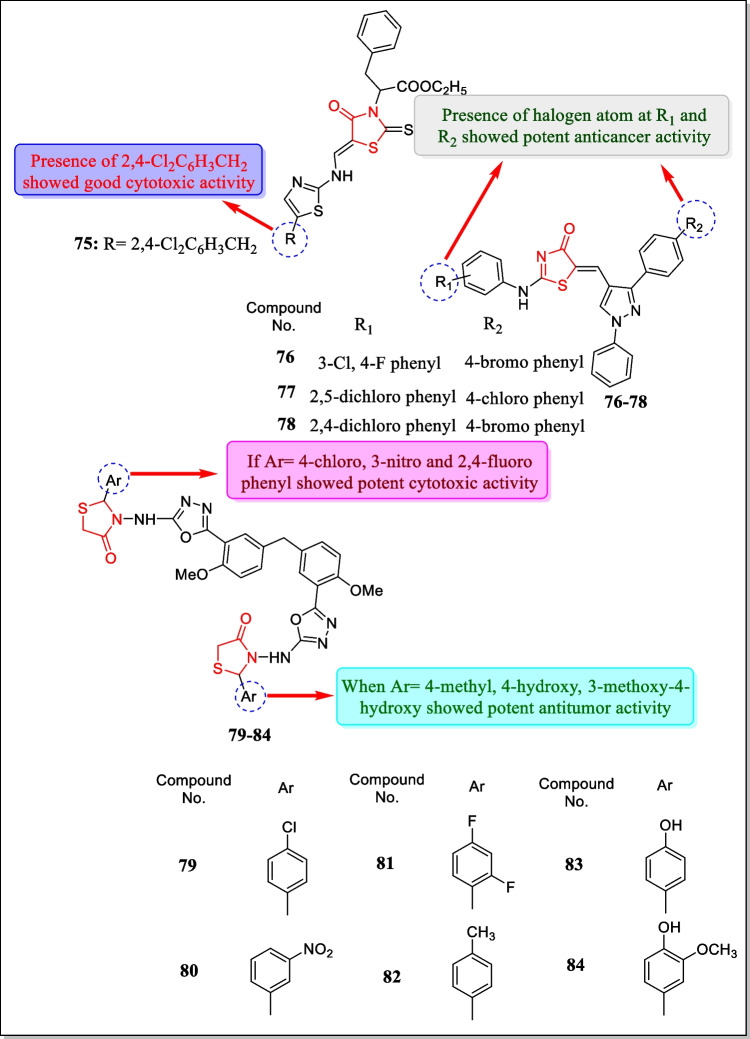
Thiazolidin-4-ones as cytotoxic agents with maximum selectivity towards different tumor cell lines

In the year 2019, Holota et al. explored a new sequence of 5-amine-4-thiazolidinone analogues and assayed for cytotoxic activity. Results identified that compound **75** (Fig. 26) exhibited strong anticancer activity in response to all 59 cancer cell lines having a GI_50_ value of 2.57 µM. Hence, the results of the study concluded that in the coming years compound **75** can be used by scientists for the invention of new anticancer medications for the treatment of cancerous diseases [179].

In 2018, the cytotoxic activity of innovative sequence of thiazolidinone-pyrazole hybrids against breast cancer cell lines EAC and MDA-MB-231 was carried out by Bhat et al. Results indicated that compound **76** showed effective anticancer activity in response to EAC cancer cell lines (IC_50_ = 901.3 µM) while **77** and **78** exhibited potent anticancer activity in response to MDA-MB-231 cancer cell lines with an IC_50_ value of 24.6 ± 2.1 µM and 29.8 ± 2.2 µM, respectively. The molecular docking study of potent compounds was performed by the Schrödinger software suite. The molecular docking study revealed that the interaction of potent compounds with HIE 96 residue of MDM2 cells showed inhibition of HIE 96 residues [180]. The structures of most potent compounds **76–78** are displyed in Fig. 26.

In 2018, a novel class of bis-heterocycles, 3-(5- [2-methoxy-5-(4-methoxy-3–5[(4-oxo-2- (aryl/hetaryl)-1, 3-thiazolan-3-yl)amino]-1, 3, 4-oxo-diazol-2-yl-benzyl)phenyl]-1, 3, 4-oxadiazol-2ylamino)-2-(2-aryl/hetaryl)-1, 3-thiazolan-4-ones were synthesized by Kumar et al. The prepared analogues were assayed for anti-proliferative activity with regard to lung cancer cell line A549 and breast cancer cell line MDA-MB-231. Among all the tested compounds, compounds **79**, **80**, and **81** exhibited potent cytotoxic activity towards A549 tumor cell line (IC_50_ = 3.59 ± 0.21, 4.22 ± 0.81, 5.19 ± 0.92 µg/mL, respectively) while compounds **82**, **83**, and **84** displayed potent cytotoxic action toward MDA-MB-231 tumor cell line (IC_50_ = 4.86 ± 0.19, 6.12 ± 0.97, 3.11 ± 1.63 µg/mL, respectively) [181]. The most potent compounds **79–84** are structurally represented in Fig. 26.

**Fig. 27:**
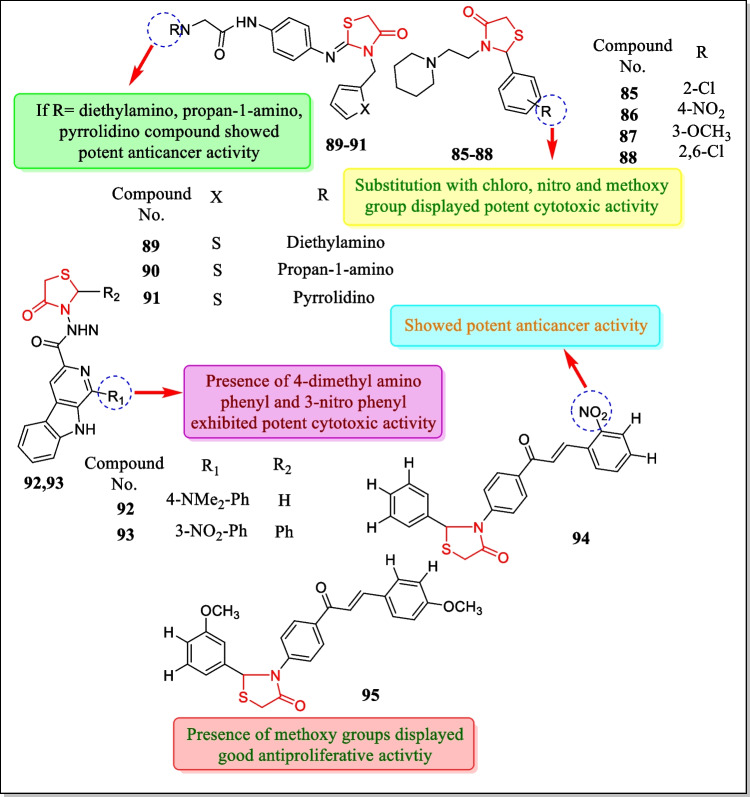
Thiazolidin-4-one containing compounds with cytotoxic activities

In the year 2017, Silveira et al. synthesized about sixteen thiazolidin-4-one derivatives and tested in vitro toward glioblastoma multiforme (an inferior form of principal brain tumor). Amidst all the tested compounds, compounds **85**, **86**, **87**, and **88** (Fig. 27) induced apoptosis through necrosis and then through apoptosis. Results demonstrated that compounds **85**, **86**, **87**, and **88** displayed 50% reduction in cell viability at a concentration of 100 µM. Treatment with sub-therapeutic doses of **85**, **86**, and **88** decreased in vivo glioma development along with cytotoxicity of embedded carcinoma such as intra-tumoral hemorrhage and peripheral pseudo-palisading [182].

In 2016, Appalanaidu et al. designed a sequence of 2-imino-4-thiazolidinone analogues and assayed towards three cancerous cell lines (line B16F10) human melanoma tumor cell, (A549) human lung cancerous cell line, (human pancreatic carcinoma cell line) PANC-1, and normal cell line (CHO) for their antitumor activity. Amidst all the screened compounds, it was found that compounds **89**, **90**, and **91** (Fig. 27) showed strong anti-cancer activity (IC_50_ = 7 ± 3.4, 3.4 ± 2, 5.4 ± 2 µM, respectively). It was also found that compounds **89**, **90**, and **91** are harmless toward non-cancerous CHO cells. Compounds **89** and **87** persuaded arrest cell cycle inG0/G1 phase while compound **90** persuaded G2/M arrest cell cycle in B16F10 cells. Compound **90** also induced intracellular ROS (reactive oxygen species) in B16F10 cells while compounds **89** and **91** showed cytotoxicity through independent ROS generation [183].

In the year 2016, Barbosa et al. scrutinized a sequence of newer hybrids of β-carboline-4-thiazolidinones and assayed for in vitro cytotoxic action in response to human carcinoma cell lines. Results concluded that compounds **92** and **93** (Fig. 27) were found most active with GI_50_ values less than 5 µM for all carcinoma cell lines. The study revealed that treatment with compound **92**(25-µM concentration) induces cell death after 15 h of treatment [184].

In 2016, Kumar et al. have described the synthesis and anti-proliferative assay of chalcone-thiazolidinone hybrids toward three tumor cell lines including A-549 (human lung tumor cell line), U-87 (human glioma cell line), and COLO-205 (human colon adenocarcinoma cell line). Out of the tested compounds, it was concluded that only analogues (**94**, **95**) (Fig. 27) were found to be the most potent on A-549 and COLO-205 (IC_50_ = 38.89 µM and 52.23 µM, respectively). Certainly, this work discovered novel molecules for the treatment of lung and colorectal cancers [185].

**Fig. 28:**
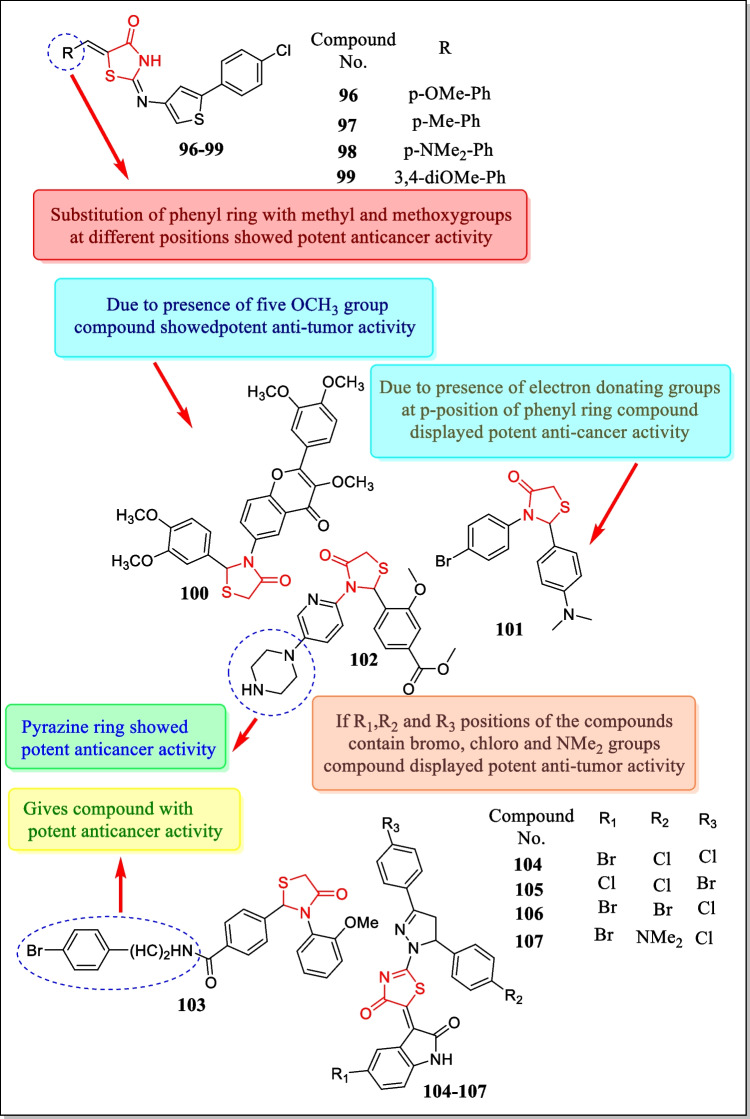
Various thiazolidin-4-one heterocycles with potent cytotoxic potential

In the year 2015, Revelant et al. announced a new class of 2-heteroarylimino-1, 3-thiazolidin-4-one analogues and assayed for (in vitro) anti-proliferative activity with regard to five human tumor cell lines HT29, HCT116, SW620, MCF-7, and MDA-MB-231. Results demonstrated that N3-substituted thiazolidin-4-ones showed conservative actions while 5-benzylidene thiazolidin-4-ones exhibited potent activities. Most potent compounds **96**, **97**, **98**, and **99** (Fig. 28) exhibited cell growth inhibition on HT29 and apoptosis in G2/M phase of the cell cycle (IC_50_ = 3.8 ± 0.9 µM, 6.4 ± 1.5 µM, 1.5 ± 1.2 µM, 10.9 ± 3.1 µM, respectively) [186].

In 2015, a novel class of thiazolidinone analogues of 6-aminoflavone was investigated and assayed for their cytotoxic activity in response to human cervical cancer cell line HeLa, melanoma tumor cell line MDA-MB-435, and kidney tumor cell lines by Moorkoth et al. Almost all the prepared analogues displayed anti-tumor activity but compound **100** (Fig. 28) showed the most potent anti-tumor activity against HeLa, MDA-MB-435, and Vero (kidney) tumor cell lines (IC_50_ = 1.19 ± 0.96, 2.36 ± 0.70, and > 100 µg/mL, respectively) [187].

In the year 2015, Sharath Kumar et al. reported a newly discovered class of 2, 3-disubstituted-4-thiazolidinone equivalents and assayed in vitro for anti-cancer activity against leukemic cell lines. Analogue **101** (Fig. 28) exhibited the promising cytotoxic activity toward blood cancer cell lines Reh cells and Nalm6 cells having IC_50_ = 11.9 and 13.5 µM, respectively [188].

In another study (2014), Sharath Kumar et al. investigated a novel class of thiazolidin-4-one analogues and assayed against human leukemic cells. One compound **102** (Fig. 28) was reported with effective antineoplastic activity towards leukemia cell lines (Nalm6, K562, Jurkat cells), and also showed apoptosis with IC_50_ values of 9.71, 15.24, and 19.29 µM, respectively [189].

In the year 2014, Wu et al. prepared a new class of 2, 3-diaryl-4-thiazolidinones and verified them for their anti-proliferative activity in response to A549 (lung cancer cell line) and MDA-MB-231 (breast cancer cell line). A large number of compounds demonstrated potent antineoplastic action towards both cancer cell lines (IC_50_ = 0.05 µM). Major findings specified that analogue **103** (Fig. 28) suppresses tumor growth metastasis and also increases the survival rate [190].

In 2014, anti-proliferative activity of ‘3-[2-(3,5-diaryl-4,5-dihydropyrazol-1- yl)-4-oxo-4,5-dihydro-1,3-thiazol-5-ylidene]-2,3-dihydro-1H-indol-2-ones’ against rat glioma cells (brain tumor cell lines) C6, Mino cells, Jurkat, and L1210 cells was carried out by Devinyak et al. Results indicated that analogues **104**, **105**, **106**, and **107** (Fig. 28) exhibited effective cytoxic action with IC_50_ value between 0.16 and 10 μM [191].

**Fig. 29:**
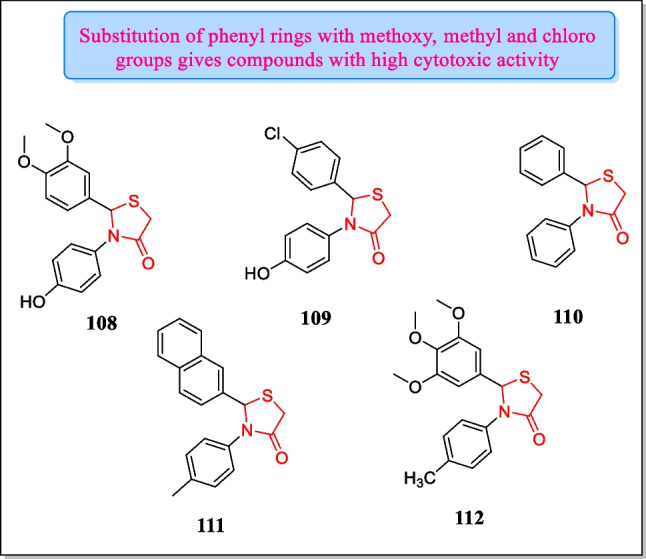
Thiazolidin-4-one bearing compounds as cytotoxic agents showing effective inhibitory action in response to MCF-7 and SKBR-3 cell lines

In 2013, Sala et al. described a sequence of 2, 3-thiazolidin-4-ones and assayed in vitro for their antineoplastic activity in response to MCF-7 and SKBR-3. The analogues **108**, **109**, **110**, **111**, and **112** (Fig. 29) exhibited advanced in vitro cytotoxic activity (IC_50_ = 2.58, 5, 0.81, 0.25, 0.23 µM, respectively). Results revealed that compound **108** showed the maximum inhibitory action towards the MCF-7 cell line while derivative **112** depicted the most potent inhibition action towards the SKBR3 cell line in contrast to other compounds [192].

**Fig. 30:**
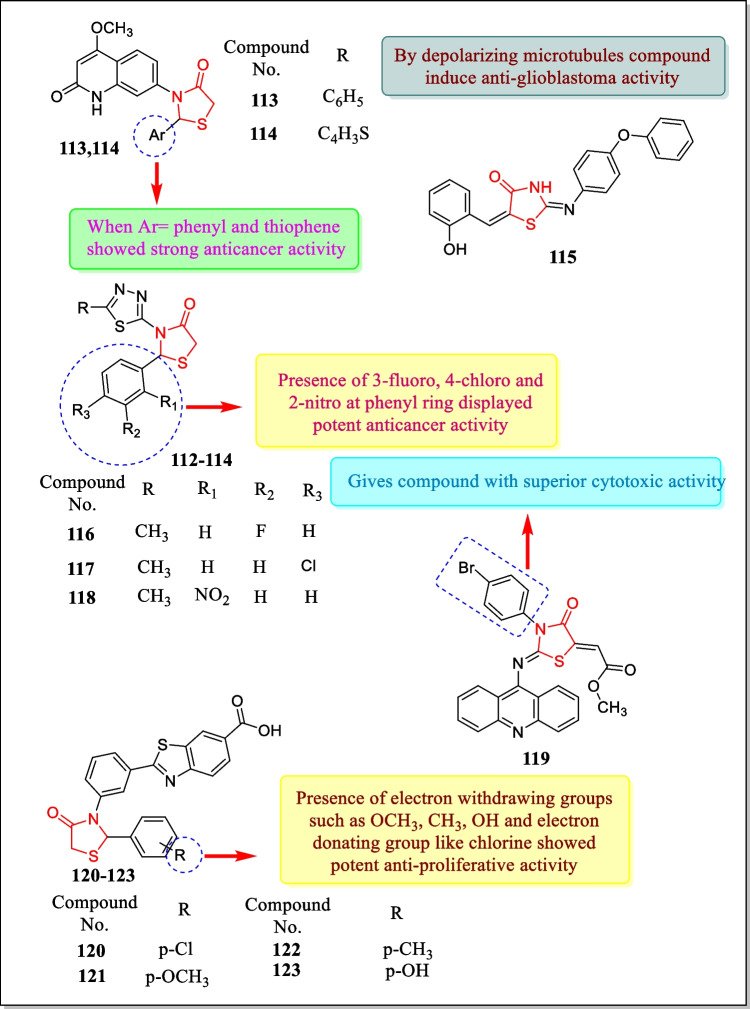
Thiazolidin-4-one analogues showed noteworthy cytotoxic action

In 2013, Suthar et al. studied the newer class of quinolone substituted thiazolidin-4-one analogues and screened for antineoplastic action toward four human carcinoma cell lines. The most active compounds **113** and **114** (Fig. 30) demonstrated strong anti-cancer activity against BT-549 (breast carcinoma cell line), HeLa (cervical carcinoma cell line), COLO-205 (colon carcinoma cell line), and ACHN renal carcinoma cell line (IC_50_ = 31.382–78.861 µM for compound **113** and 20.737–44.711 µM for compound **114**, respectively) [193].

In 2013, Zhang et al. reported a synthesis of ‘(2E, 5Z)-5-(2-hydroxybenzylidene)-2-((4-phenoxyphenyl)imino)thiazolidin-4-one’ derivatives and screened for its glioblastoma (brain tumor) against U87MG human glioblastoma cells. Results proved that compound **115** (configurationally evidenced in Fig. 30) exhibited anti-proliferative action in response to U87MG human glioblastoma cell having an IC_50_ value of 20 µM. Results also demonstrated that compound **115** arrests cell growth at the G2/M phase and also convinced apoptosis [194].

In 2013, a sequence of 5-alkyl/aryl thiadiazole substituted thiazolidin-4-one derivatives were prepared and assessed for anti-proliferative action in response to breast cancinoma cell line MCF-7 by Joseph et al. Results indicated that almost all the analogues displayed good anti-tumor activity with IC_50_ less than 150 µmol L^−1^. Compounds **116**, **117**, and **118** (Fig. 30) were found effective among all the tested compounds (IC_50_ = 66.84, 60.71, and 46.34 µM) [195].

In 2012, Paulikovà et al. designed thiazolidin-4-one analogues and confirmed for their antineoplastic activity towards a number of leukemic cell lines, HL-60, L1210, and A2780. Results proved that compound **119** (Fig. 30) showed strong cytotoxic activity having IC_50_ values of 1.3 ± 0.2, 3.1 ± 0.4, and 7.7 ± 0.5 µM, respectively. This compound repressed the proliferation of the cells and also tempted cell death [196].

In 2012, Prabhu et al. prepared a novel class of 2-phenyl thiazolidinone substituted 2-phenyl benzothiazole-6-carboxylic acid analogues and investigated for in vitro anti-neoplastic action towards HeLa. Analogues **120**, **121**, **122**, and **123** (Fig. 30) exhibited anti-cancer activity in regard to HeLa cervical cell lines (IC_50_ = 9.768, 14.566, 16.456, 17.768 µg/mL). Compound **120** was found most potent as compared to **121**, **122**, and **123** [197].

**Fig. 31:**
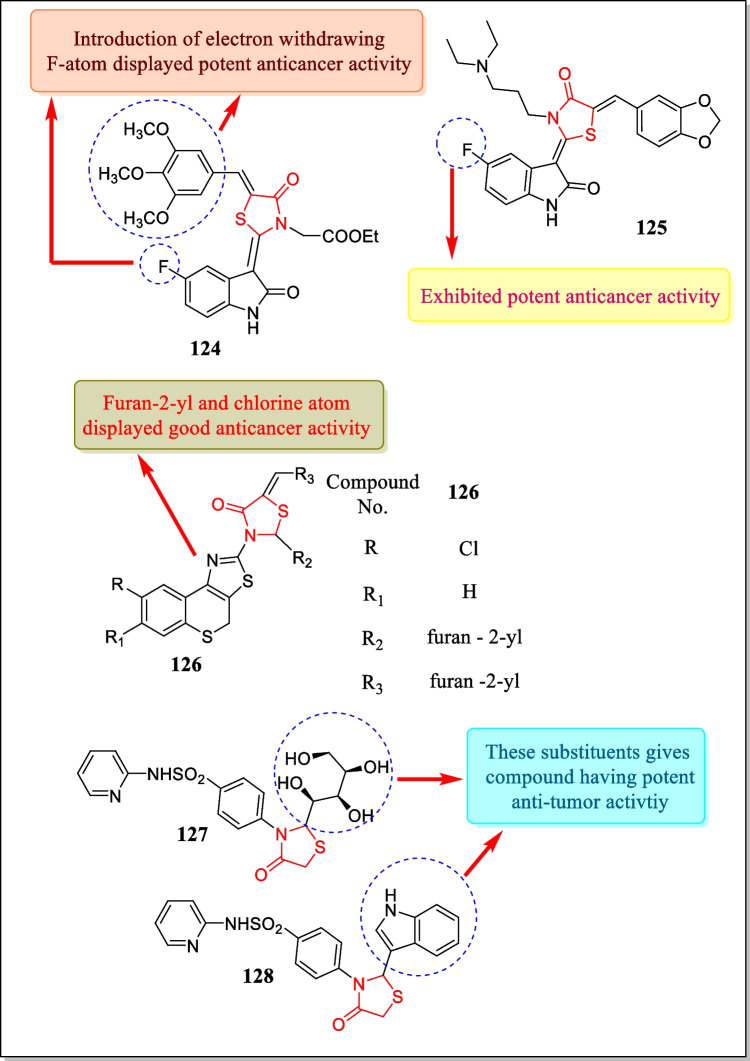
Thiazolidin-4-one bearing compounds as potent anticancer agents

In 2012, Wang et al. reported a novel sequence of indolin-2-one analogues having thiazolidin-4-one nucleus and investigated them for antitumor activity towards three tumor cell lines, HT-29 (human colon cancer cell line), H460 (human lung cancer cell line), and MDA-MB-231 (human breast cancer cell line) by standard MTT assay. Several prepared analogues showed noteworthy cytotoxicity. Few potent analogues were further assayed toward the (WI-38) cell line. Among the synthesized analogues, analogue **124** (Fig. 31) was found to have the most powerful anticancer action in response to HT-29, H460 and MDA-MB-231 cell lines with IC_50_ values of 0.016 µmol/L, 0.0037 µmol/L, and 10.5 µmol/L, respectively. Major findings from the study indicated that if the 2^nd^ position of thiazolidin-4-one ring substituted with the mixture of the indolin-2-one core structure and 5-benzylidene-4-thiazolidinone nucleus, it could improve the anticancer activities [198].

In 2011, the cytotoxicity of innovative indolin-2-one and thiazolidin-4-one analogues against HT-29 (human colon cancer), MDA-MB-231 (human breast cancer), SMMC-7721 (human liver cancer), and H460 human lung cancer cell lines was presented by Wang et al. Major findings concluded that compound **125** (Fig. 31) displayed potent anti-cancer activity towards HT-29, H460, MDA-MB-231, and SMMC-7721 cell lines possessing IC_50_ values of 0.025, 0.075, 0.77, and 1.95 mM, respectively [199].

In 2011, Ma et al. designed the microwave-assisted synthesis of thiazolidinones by three component reactions using aromatic aldehyde, 2-amino-4*H*-benzothiopyrano [4, 3-*d*] thiazole, and mercaptoacetic acid. All the synthesized analogues were screened for their antineoplastic action by in vitro methods against various cancinoma cell lines by using the MTT assay technique. Out of all manufactured analogues, analogue **126** (Fig. 31) displayed the effective anticancer activity (IC_50_ = 5.76 μM, 6.19 μM, 6.67 μM, respectively) toward SGC-7901 (gastric cancer cells), Hela (cervical cancer cells), and A-549 (lung cancer cells), respectively [200].

In 2010, Kamel et al. identified an innovative succession of various compounds with thiazolidinone derivatives and assayed for their anti-proliferative activity in response to MCF7 (breast cancer cells) and HeLa (cervical cancer cells). Among the thiazolidinone derivatives, analogues **127** and **128** were displayed potent anti-proliferative activity in response to cell lines MCF7 and HeLa with IC_50_ values of 2.68 and 2.01 µg/mL for MCF7 tumor cell line and 1.07 and 1.95 µg/mL for HeLa tumor cell line respectively [201]. The structures of the analogues **127** and **128** are stated in Fig. 31.

**Fig. 32:**
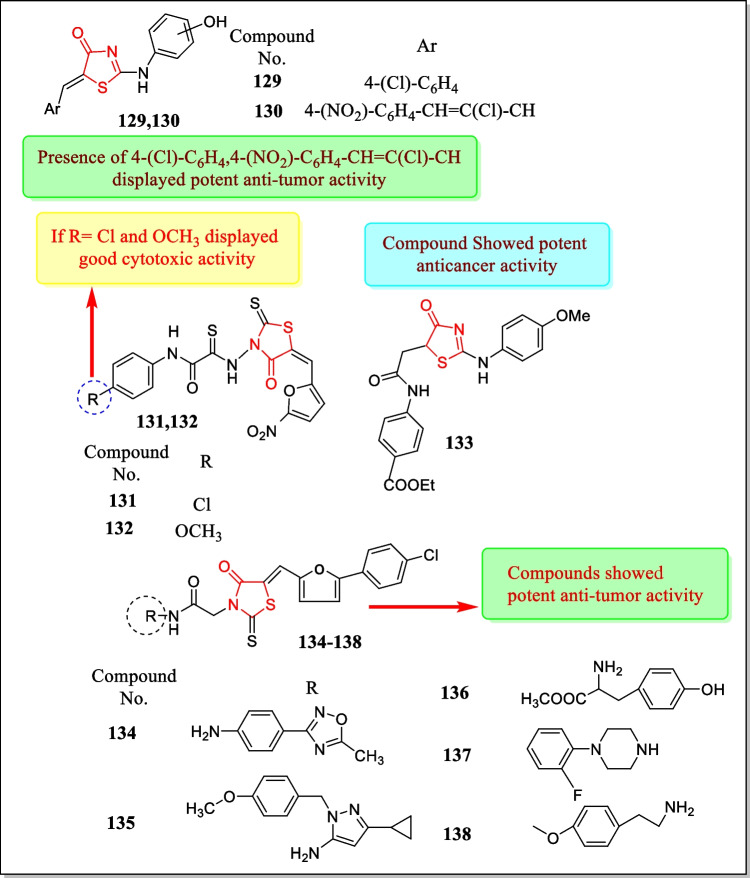
Compounds bearing thiazolidin-4-one scaffold as promising cytotoxic agents

In 2010, a sequence of new 5-arylidene-2-arylaminothiazol-4(5*H*)-ones and 2-aryl(benzyl)amino-*1H*-imidazol-4(*5H*)-ones derivatives have been testified by Subtel’na et al. Each manufactured analogue was investigated for in vitro anticancer action in response to 60 tumor cell lines. Most of the analogues unveiled encouraging cytotoxic effects at micromolar and submicromolar levels (mean Log GI_50_ ranges − 5.77 to − 4.35). Compounds **129** and **130** (Fig. 32) exhibited the maximum activity, contrary to leukemia cell lines at the submicromolar level having mean Log GI_50_ values − 6.41 and − 6.29, respectively [202].

In 2010, Ramkumar et al. synthesized several 2-thioxo-4-thiazolidinones (rhodanine) containing analogues as 2^nd^-generation IN inhibitors from the previously identified compounds through structural modifications to overcome the resistance acquired by some viral strains. These compounds were then screened for evaluation of their selectivity towards human a purinic/pyrimidinic endonuclease 1 (APE1) and also assessed for cytotoxic potential. Compounds **131** and **132** showed interesting activity in response to colon cancer cell line HCT 116 (GI_50_ =  > 10 for compound **131**, 5.1 ± 0.8 µM for compound **132** against HCT 116 p53^+/+^, GI_50_ = 6.5 ± 1.2 for compound **131**, 6.1 ± 2.2 µM for compound **132** against HCT 116 p53^−/−^) and pancreatic cell line Panc-1 (GI_50_ = 8.6 ± 1.6 µM for compound **131**, 8.2 ± 1.2 µM for compound **132**). Major findings indicated that rhodanines act as prime for the expansion of newer cytotoxic agents [203]. The most potent analogues are structurally designed in Fig. 32.

In another study (2010), Subtel’na et al. arranged a sequence of ‘5-substituted-2-(4-alkoxyphenylamino)thiazol-4(5*H*)-one’ analogues and assessed them for antitumor activity in response to a panel of sixty human tumor cell lines. Outcomes of the study demonstrated that 5-carboxymethyl-2-(4-methoxyphenylamino)-thiazole-4(5*H*)-one **133** (Fig. 32) (IgGI_50_ =  <  − 5.50 µM, lgTGI =  ≤  − 5.00 µM, lgLC_50_ = ˂ − 4.50 µM) exhibits potent antitumor activity and this compound was selected as the major compound for further studies and shows high cytotoxic probable in response to CNS cancer and melanoma [204].

In 2010, Chandrappa et al. reported a group of new thioxothiazolidin-4-one analogues. All the synthesized analogues were assessed for their capability to inhibit tumor growth and angiogenesis brought by tumor by means of mouse EAT (Ehrlich ascites tumor) as a model system. Compounds **134** to **138** (Fig. 32) exhibited significant anticancer activity and can be evaluated for anticancer therapy owing to their tumor angiogenesis and cell proliferation inhibitory potential. Compounds **134**, **135**, and **138** indicated mean ascites volumes of 5.2 ± 0.68 mL, 4.8 ± 0.49 mL, and 5.7 ± 1.26 mL, respectively [205].

**Fig. 33:**
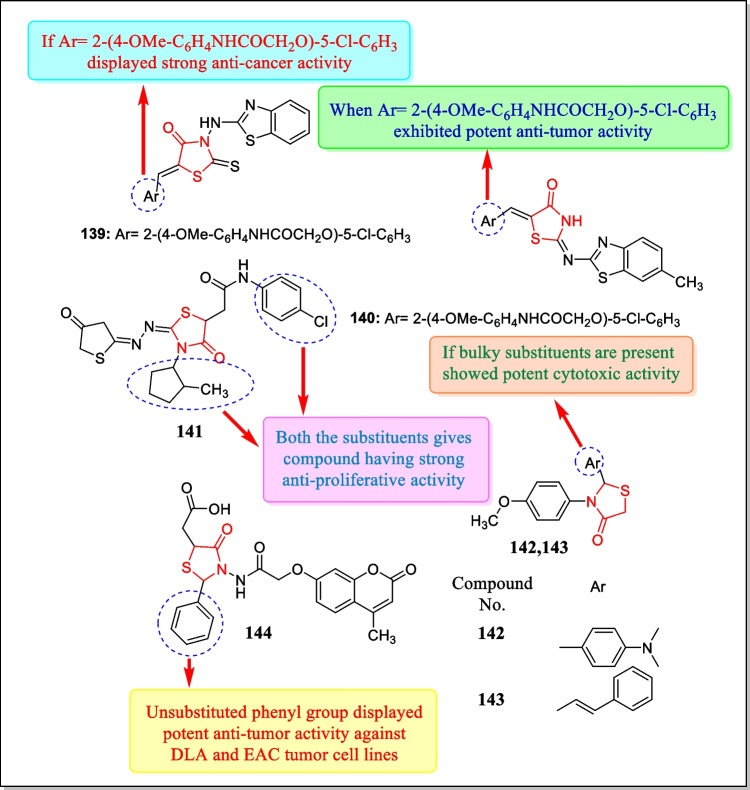
Analogues containing thiazolidin-4-one scaffold as anti-proliferative agents

In 2010, Havrylyuk et al. presented a new array of thiazolidin-4-one analogues containing benzothiazole moiety and assayed for anti-proliferative activity by the National Cancer Institute. Compounds **139** and **140** (Fig. 33) exhibited anti-tumor activity against colon, renal, melanoma, prostate, ovarian, CNS, breast, and lung tumor cell lines. The most active analogue **139** showed strong anti-cancer activity with logGI_50_ and logGI values of − 5.38 and − 4.45 µM, respectively [58].

In another study (2009), Havrylyuk et al. reported some novel non-fused bicyclic thiazolidin-4-one derivatives. About eight synthesized analogues were assayed for anti-proliferative activity and three analogues showed a different level of anti-cancer activity. Results demonstrated that compound **141** (Fig. 33) was the most effective analogue and exhibited antineoplastic activity, contrary to melanoma, CNS, renal, breast, leukemia, colon, ovarian, lung, and prostate tumor cell lines having a mean logGI_50_ and logTGI values of − 5.35 and − 4.78 µM, respectively [59].

In 2009, Jubie et al. prepared a new sequence of 3-(methoxyphenyl)-2-aryl thiazolidin-4-ones through the reaction of mercaptoacetic acid with Schiff’s bases of 4-methoxy aniline. Analogues have been confirmed for cytotoxicity by SRB assay method in (human epithelial type-2) Hep-2cell line. Two compounds **142** and **143** (Fig. 33) depicted potent cytotoxic activity (CT_50_ = 62 and 45 µg/mL) [206].

In 2009, Kumar et al. designed a few coumarinyl heterocycles and also described their potential role as cytotoxic mediators toward Dalton’s lymphoma ascites cancer cells (DLA) and Ehrlich ascites carcinoma cells (EAC). Few synthesized derivatives exhibited good in vitro antioxidant and cytotoxic activity. The result indicated that compound **144** (Fig. 33) at 0.1 mmol L^–1^ concentration exhibited comparable free radical scavenging activity (95%) toward standard ascorbic acid. Cytotoxicity studies showed that compound **144** was found to be a noble cytotoxic mediator in response to DLA cells at a concentration of 100 mg mL^–1^ and to be effective cytotoxic agents in response to EAC cells at a concentration of 50 mg mL^−1^ [207].

**Fig. 34:**
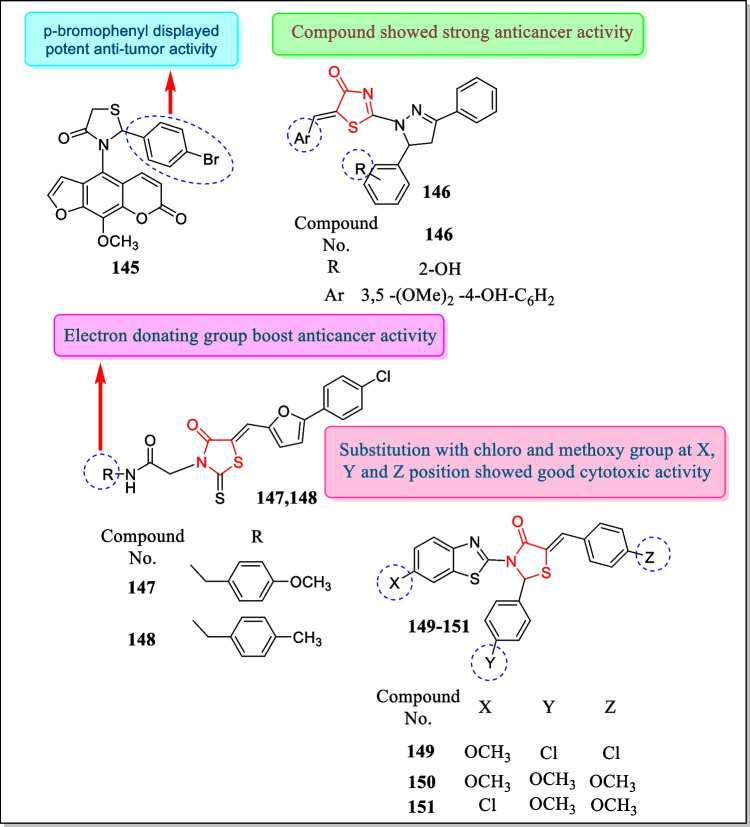
Thiazolidin-4-one comprising analogues as effective antitumor agents

In 2009, some new xanthotoxin analogues were arranged and assayed for their cytotoxic potential in response to HeLa (cervical cancer cells) and MCF-7 (breast cancer cells) by Hafez et al. Among all the produced analogues, five compounds demonstrated activity in preventing the growth of HeLa cells. The outcomes concluded that compound **145** (Fig. 34) (IC_50_ = 40 µg/mL) was found to have substantial anticancer activity in response to cancerous cell lines [208].

In 2009, several novel pyrazoline substituted thiazolones have been manufactured by Havrylyuk et al. The prepared derivatives were testified for in vitro cytotoxic potential in response to lung, colon, leukemia, melanoma, ovarian, CNS, prostate, and renal, along with breast, cancerous cell lines. The prepared analogue’s structures were ascertained based on data obtained by analysis employing NMR, LC–MS, and X-ray technique. Among the synthesized compounds, analogue **146** (Fig. 34) was found most active towards colon cancer cell lines with average log GI_50_ and log TGI − 5.61 and − 5.14, respectively [209].

In 2009, Chandrappa et al. prepared several 2-(5-((5-(4-chlorophenyl) furan-2-yl) methylene)-4-oxo-2-thioxothiazolidin-3-yl) acetic acid analogues and screened for cytotoxic potential toward human leukemia cell lines. Out of all the produced analogues, analogues **147** and **148** were found to be the most active (IC_50_ = 40 ± 2, 45 ± 2 µM in response to k562 tumor cell line, 44 ± 2, 45 ± 3 µM against Reh cell line, respectively). The major findings indicated that thiazolidinone analogues with an electron-releasing group at the fourth site of the aryl ring demonstrated better anticancer action [210] (Fig. 34).

In 2009, Shashank et al. reported some novel benzothiazole derivatives having thiazolidinone moiety. All synthesized analogues were assayed for their antineoplastic activities by MTT assay against melanoma cancer cell M-14, human normal epithelial breast cancer cell HLB-100, and human colon cancer cell SW-620. Analogue **149** was found most effective in response to M-14 cancer cell (IC_50_ = 54.15 ± 0.152 μg/mL), compound **150** showed highest activities against HLB-100 cancer cell having IC_50_ value 126.15 ± 0.4454 μg/mL, and compound **151** was found most potent against SW-620 cancer cell (IC_50_ = 49.23 ± 0.593 μg/mL). Major findings from the study concluded that analogues possessing chloro group at *p*-position showed better antitumor activity [211]. The structures of analogues **149–151** are shown in Fig. 34.

**Fig. 35:**
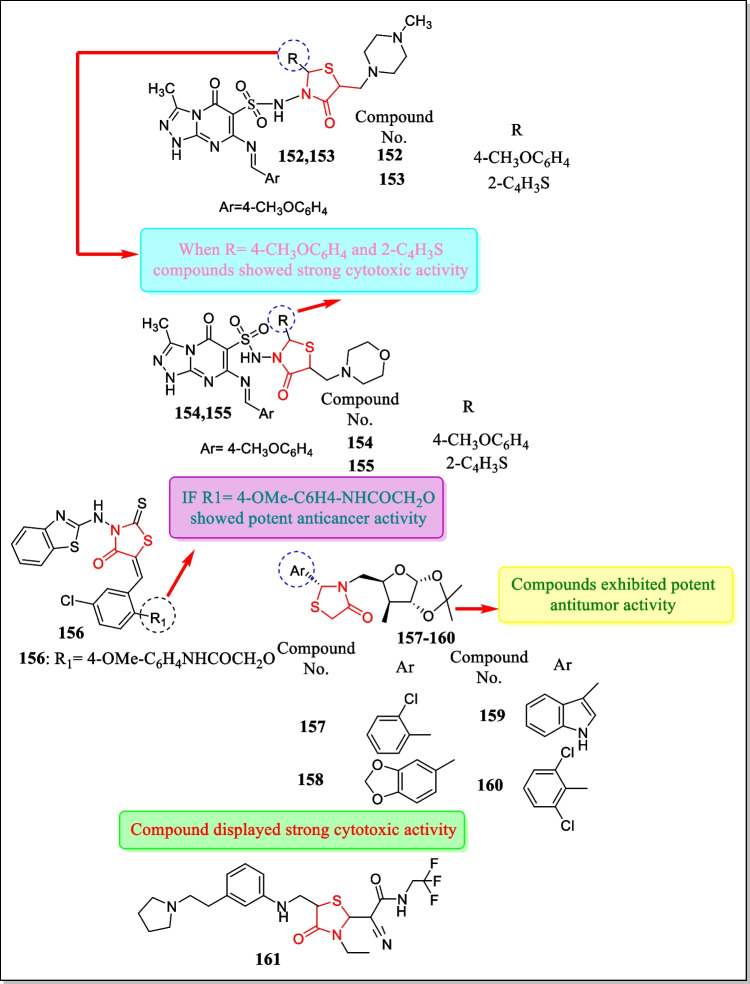
Some thiazolidin-4-one containing analogues as cytotoxic agents

In the year 2009, Hafez et al. designed and evaluated thiazolidin-4-one moiety containing triazole [4, 3-α] pyrimidin-6-sulfonamide derivatives for anti-malignant potential against several carcinoma cell lines. The most active analogues 152, 153, 154, and 155 (Fig. 35) demonstrated strong anti-proliferative activity against almost all carcinoma cell lines at 10^–5^ µM and in few cases at 10^–7^ µM concentrations. Compound **152** showed momentous growth inhibition of all sixty cell lines (GI_50_ 5.89–37.1 × 10^–6^ µM) and growth inhibition of 56 cell lines at 2.1–8.94 × 10^–5^ M and cytotoxic activity on 31 cell lines. Compound **153** at 0.318–9.73 × 10^–5^ µM concentration showed inhibition of growth of twenty cell lines and anti-malignant activity on three leukemia cell lines (HL-60 TB, K-562, and SR). Compound **154** with LC_50_ values between 7.15 and 8.68 × 10^–5^ showed cytotoxicity on four leukemia cell lines. Fifty percent growth of 26 cell lines was repressed by compound **155** at micromolar concentrations while inhibiting total growth of 26 cell lines at 10^–5^ M concentration and also displayed effective activity in response to leukemia SR cell lines (TGI = 3.18 × 10^–6^ µM and LC_50_ = 9.20 × 10^–6^ µM). Compound **155** also exhibited anti-malignant activity towards colon cancer cell lines HCC-2998 having an LC_50_ value of 6.48 × 10^–5^ µM [212].

In 2009, a new array of 2-thioxo-thiazolidin-4-ones with benzothiazole analogues was identified by Mosula et al. All the synthesized analogues were assayed in response to various tumor cell lines including non-small cell lung tumor, renal cancer, and ovarian tumor cell lines. Among examined compounds, compound **156** (Fig. 35) disclosed the maximum anticancer activity (logGI_50_ and logTGI =  − 5.38 and − 4.45 µM) [213].

In 2008, Chen et al. synthesized some new ‘2-aryl-3-[5-deoxy-1, 2-O-isopropylidene-α-D-xylofuranose-5-C-yl] thiazolidin-4-ones.’ The structures of the new moieties were confirmed by NMR (nuclear magnetic resonance) spectroscopy, mass spectrometry (MS), and X-ray crystallography analysis. Glycosidase inhibition and antitumor activities of such compounds toward human cervical cancer cell line HeLa were preliminarily evaluated. Some of the analogues, **157** to **160** (Fig. 35), exhibited antitumor activity with % inhibition of 31.8, 28.0, 20.5, 20.2, at 100 µM, respectively [214].

In 2007, Santamaria et al. described the innovation and application of zk-thiazolidinone (TAL) **161** as a potent small-molecule inhibitor of mammalian polo-like kinase 1 (Plk_1_) which is a significant mitotic progression regulator and cell division and has been well thought out as a probable target for cancer treatment. As (TAL) **161** (Fig. 35) was tested against a panel of 93 serine/threonine and tyrosine kinases, it was found to be highly selective in inhibiting human Plk1 with IC_50_ value of 19 ± 12 nM. Major findings from the study concluded that thiazolidinones signify a new chemical class of kinase inhibitors [215].

**Fig. 36:**
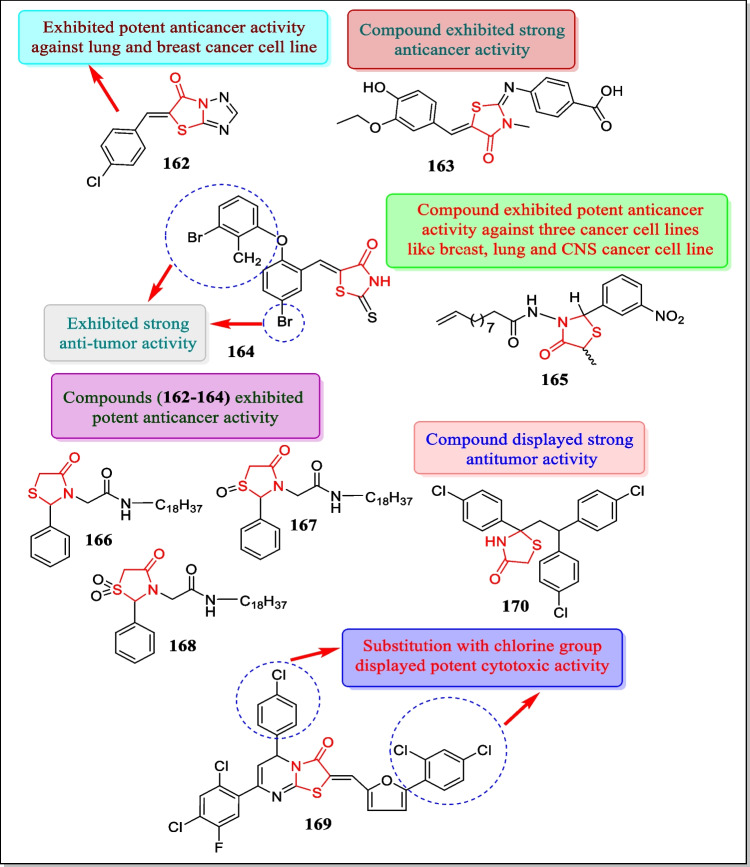
Thiazolidin-4-one analogues as cytotoxic agents showed effective potential toward various tumor cell lines

In 2007, a novel series of 5-substituted thiazolo [3, 2-b][1,2,4]triazol-6-ones analogues was reported by Lesyk et al. The structural characterization of the prepared analogues was done by appropriate methods such as ^1^H NMR, ^13^C NMR, and X-ray analysis. Synthesized analogues were confirmed for antineoplastic potential on a panel of nearly 60 human cancer cell lines. Among them, derivative **162** (Fig. 36) displayed the most significant action with a discriminating effect on leukemia cell lines having logGI_50_/logTGI of − 5.08/ − 4.47 to − 6.45/ − 4.70 µM [216].

Structurally, integrins are heterodimeric receptors having an α and αβ subunit, which bind in a noncovalent manner in defined combinations. An important role has been found in tumor progression and metasis involving integrin α_v_*β*_3_ receptors. So, this receptor is a tremendous target for anticancer drugs. In 2006, a series of novel α_v_β_3_ antagonists has been discovered by Dayamet al*.* through a database of small-molecule drug-like analogues using pharmacophore screening. Based on structural specificity, twenty-nine analogues were selected for the screening of binding assay ofthe α_v_β_3_ receptor and four analogues were found to have a strong binding affinity. Compound **163** (Fig. 36) was found most active having an IC_50_ value of 0.34 µM toward human melanoma cell line MDA-MB-435, 13 ± 8 µM toward human ovarian adenocarcinoma cell line HEY, > 20 µM toward human breast tumor cell line MCF-7, and > 20 µM toward NH3T3 tumor cell line [217].

In 2006, a series of rhodanine moieties were explored by Ahn et al*.* The prepared analogues were observed in vitro for inhibitory activity toward recombinant human PRL-3. PRL proteins promote cell motility, invasion action, and metastasis, which are directly reliant on their catalytic action; thus, PRL-3 acts as an important therapeutic target for metastastic tumors. Out of all synthesized compounds, compound **164** (Fig. 36) displayed a potent analogue with an IC_50_ value of 0.9 µM [218].

In 2005, Rahman et al. designed innovative long alkyl chain substituted 4-thiazolidinone and thiazan-4-one derivatives. Out of the prepared analogues, few moieties were designated by the National Cancer Institute (NCI) and verified them for anti-malignant action in contrast to sixty cell lines of human cancers (nine types). From the study, it was concluded that analogue **165** (Fig. 36) was found to have momentous anti-malignant potential. For lung, melanoma, and renal tumors, the compound shows a decrease in growth of 75%, 97%, and 84%, respectively, at the concentration of 1.0 × 10^–4^ M [219].

In another study (2004), Gududuru et al. synthesized asequence of 2-aryl-4-oxo-thiazolidin-3-yl amides intending to enhance the selectivity and improve the potency as compared to serine amide phosphates (SAPs) as a cytotoxic agent for prostate tumor. The synthesized analogue’s cytotoxic potential was screened in response to five human prostate melanoma cell lines (DU-145, PC-3, LNCaP, PPC-1, and TSU), and in RH7777 cells (negative controls). 5-Fluorouracil was utilized as a reference medication. Amidst all of the prepared moieties, three compounds **166**, **167**, and **168** have shown the most potent activity (IC_50_ = 7.1–39.6 µM for compound **166**, 5.4–11.5 µM for compound **167**, 5.5–22.1 µM for compound **168**) with improved selectivity as compared to SAPs [220]. The structures of compounds **166–168** are shown in Fig. 36.

In 2004, Holla et al. prepared 2-(5-arylfurfurylidene/5-nitrofurfurylidene)-5-aryl-7-(2,4-dichloro-5-fluorophenyl)-5*H*-thiazolo[2,3-*b*]-pyrimidin-2(1*H*)-ones. The analogues were found to have moderate to excellent inhibitory activity toward a panel of sixty cell lines of different cancers. Compound **169** (Fig. 36) depicted maximum activity in response to leukemia HL-60 (TB) cell line (GI_50_ = ˂0.001 µM) [221].

In 2002, Singh et al. synthesized novel ‘2-(2, 2-bis (4-chlorophenyl) ethyl]-2-(4-chlorophenyl)-thiazolidin-4-ones.’ Compound **170** (Fig. 36) was screened at five dissimilar concentrations towards sixty cell lines of nine types of human tumors: namely, ovarian, lung, renal, melanoma, leukemia, colon, CNS, prostate, and breast for cytotoxic activity. The newly synthesized compounds depicted antitumor activity only at higher concentrations and cause a reduction in the growth of melanoma, colon, and renal cancer cells 52, 80, and 91%, respectively. Compound **170** demonstrated potent antitumor activity with log GI_50_ value of 0.75–5.67 µM, respectively [222].

### Key structural feartures of thiazolidinones responsible for potent anticancer activity

**Fig. 37:**
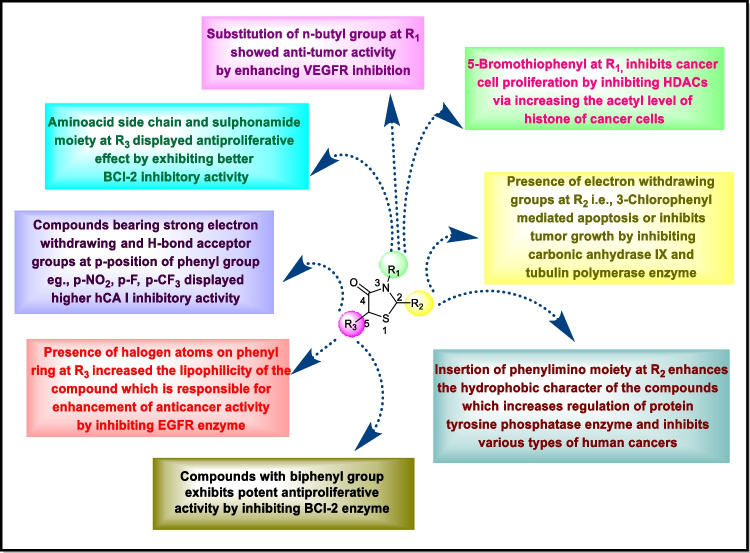
Important structural features of thiazolidinone derivatives

From the description presented in this manuscript and from critical analysis of the scientific information contained herein, the following key structural features have been found pivotal in determining anticancer activity of thiazolidin-4-one derivatives. The important strututral features are (Fig. 37):

- Substitution of 5-bromophenyl, n-butyl group, and amino acids along with sulfonamide moiety at R_1_ exhibits anticancer activity via inhibition of HDAC, VEGFR, and BCl-2 enzymes
- Compounds containing electron withdrawing groups, i.e., 3-chlorophenyl moiety at R_2_ inhibits carbonic anhydrase IX, tubulin polymerase enzyme, and protein tyrosin phosphates to regulate cancer development.
- Presence of phenylimino moiety at R_2_ enhances the hydrophobic character of the compounds which increases regulation of protein tyrosine phosphatase enzyme and inhibits various types of human cancers
- When R_3_ position substituted with electron withdrawing and H-bond acceptor groups, i.e., p-NO_2_, p-F, and p-CF_3_ moieties displayed anti-tumor activity by hindering carbonic anhydrase I enzyme
- Adding halogen atoms to the phenyl ring at position R3 made the chemical more lipophilic, which enhanced its anticancer potential by inhibiting the EGFR enzyme
- Presence of amino acid side chain and sulfonamide moiety at R_3_ displayed antiproliferative effect by exhibiting better BCl-2 inhibitory activity

## Various patents filed/granted on thiazolidin-4-one derivatives as anticancer agents

In organic chemistry, the expansion of new synthetic methodologies giving structures which combine numerous pharmacologically active molecules into a single molecule bears attracted considerable interest. In this direction, heterocyclic compounds occupy a superior place among pharmaceutically active compounds and the production of a simple and effective synthesis of analogues containing multi-heterocyclic ring has given the drug discovery new aspects. Hundreds of patents in the field of thiazolidinones with a diverse diversity of biological activities have been published over the last few decades. This paper is an intense effort to accumulate the key patents filed or granted to thiazolidinone analogues as anticancer agents with an aim that the data delivered in paper will be beneficial for future research in this field (Table 1) [223].

**Table 1:** Various patents filed or granted to thiazolidinone analogues as anticancer agents

Sr. No	Date	Patent No	Invention disclosed	References
1	24–04-2018	US 9, 949, 961 B2	Agents for the prophylaxis and/or treatment neoplastic diseases	[224]
2	13–06-2017	US 9, 675, 596 B2	1,2,4-Thiazolidin-3-one derivatives and their use in the treatment of cancer	[225]
3	20–10-2015	US 9, 162, 994 B2	1,2,4-Thiazolidin-3-one derivatives and their use in the treatment of cancer	[226]
4	21–02-2012	US 8,119,812 B2	The compounds of the invention inhibited the CDC7 protein kinase activity, and suppression of cell proliferation	[227]
5	21–07-2011	US 2011/0177046 A1	Dithiazolidine and thiazolidine derivatives as anticancer agents	[228]
6	04–08-2011	US 2,010,190,299 A1	Novel thiazolidinone derivative having a CDC7 inhibitory action	[229]
7	18–05-2010	US 7, 718, 681 B2	5-(1,3-Diaryl-1H-pyrazol-4-yl methylene)-thiazolidin-2,4-dione derivatives useful as anticancer agent	[230]
8	16–02-2010	US 7, 662, 842 B2	Thiazolidinone amides, thiazolidine carboxylic acid amides, and serine amides, including polyamine conjugates thereof as selective anticancer agents	[231]
9	11–11-2007	US 7, 307, 093 B2	Thiazolidinone amides, thiazolidine carboxylic acid amides, methods of making and use thereof	[232]
10	16–10-2008	US 2008/0255213 A1	Thiazolidinone amides, thiazolidine carboxylic acid amides, and serine amides, including polyamine conjugates thereof, as selective anticancer agents	[233]
11	23–10-2008	US 2008/0261,980 A1	The invention describes the use of compounds for the preparation of pharmaceutical compositions for the treatment of pathologies in which inhibition of the interaction between HIF-1α and p300 is beneficial, in particular as antiangiogenic medicaments for the therapy of solid tumors	[234]
12	11–01-2007	US 2007/0010565 A1	New thiazolidinones without basic nitrogen, their production, and use as pharmaceutical agents	[235]
13	13–04-2006	US 0,079,503 A1	Thiazolidinones and the use thereof as polo-like kinase inhibitors	[236]
14	20–05-2004	US 2004/0097566 A1	2-Substituted thiazolidinone and oxazolidinone derivatives for the inhibition of phosphatases and the treatment of cancer	[237]

## Future perspectives

As heterocyclic chemistry is the emerging field which promotes readily selectable interactions with the biological targets and resulting in a combination of structural and metabolic stability. In recent years with considerable efforts, several scientists looked upon in development of novel heterocyclic compounds of biological interest. The growing danger of drug resistance against cancer cells makes the urgent need to develop novel heterocyclic moieties with promising anticancer activity. Numerous thiazolidinone derivatives have been used for a few years to cure cancer. However, a few of the analogues exhibited less inhibitory action in response to cancer cells and were also linked to a few undesirable side effects. Moreover, drug-sensitive and drug-resistant tumor cells are making the cancer much worse. Therefore, the SAR method is thought to be the best for improving their selectivity towards cancer cells by substituting the thiazolidinone core structure with efficient substitutions. Some nanoparticle-based conjugation can also bring better targeted effects against cancer. As per recent technological approach, a number of heterocyclic compounds has been developed with potent anticancer activity. As therapeutically active pharmacophores, thiazolidin-4-one has a myriad of applications in development of novel derivatives against various cancer cell lines. Thiazolidin-4-one has become one of the prime drug research scaffold and its analogues have proved to be desirable compounds because of their exceptional pharmacological activities. Hence, the research inceptions will persist decisive to the innovation of such lead compounds to meet all the required criteria both pharmacologically and pharmacokinetically for development of potent anticancer medications. As the above study reveals, derivatives based on thiazolidin-4-one core nucleus are likely to be considered as several target enzyme inhibitors and mentioned downsides should also be taken into consideration in future research development of more potent thiazoldinones for oncotherapy.

## Conclusion

Thiazolidin-4-one derivatives possess an important and unique place in the medicinal chemistry of antineoplastic agents. Their derivatives have been reported to demonstrate promising anticancer activities in the recent scientific literature. A great volume of research has been carried out for the progression of new and effective anticancer agents from these heterocyclic scaffolds. Expansion of novel synthetic strategies for the production of newer compounds, structural characterization, and evaluation of biological potential is gaining increasing attention of the scientific community across the globe. The current review highlights new horizons of thiazolidin-4-one–based heterocyclic derivatives with anticancer potential and chemical aspects. A detailed discussion on the structural activity relationship studies of thiazolidin-4-ones as possible multi-target enzyme inhibitors and possible synthesis through click chemistry and green chemistry for the developments of future promising thiazolidin-4-one-containing compounds has also been provided. Several patents granted on 4-thiazolidinone derivatives related to anticancer activity have also been presented in tabular form. The scientific data compiled herein indicates that these compounds have promising potential as possible anticancer agents. It is predicted that scientific information enclosed in this paper might be suitable for the forthcoming researchers functioning on this heterocyclic scaffold as possible anticancer agents. Information compiled in this paper can be helpful for medicinal chemists and other researchers for further exploration of these heterocyclic compounds as possible anticancer agents.

## Abbreviations

DNA: Deoxyribonucleic acid
COX: Cyclooxygenase
LOX: Lipooxygenase
DMF: Dimethylformamide
CDK: Cyclin-dependent kinase
GSK3β: Glycogen synthase kinase 3β
CRAF: Cellular rapidly accelerated fibrosarcoma
ERBB: EGF receptor family
FLT: FMS-like tyrosine kinase
PIM: Proviral Integrations of Moloney virus 2
C-Met: Multi-tyrosine kinase
Akt: Serine/threonine-protein kinase
Src: Non-receptor tyrosine-protein kinase
Ron: Receptor tyrosine kinase
KDR: Kinase insert domain receptor
EGFR: Epidermal growth factor receptor
C-Kit: Receptor tyrosine kinase
IGF-1R: Insulin-like growth factor 1 receptor
EAC: Ehrlich ascites carcinoma
*K*_i_: Rate of cell growth
HER-2: Human epidermal growth factor receptor
Bcl-2: B-cell lymphoma 2
AXL: Receptor tyrosine kinase
ALK: Anaplastic lymphoma kinase
µM: Micromolar
nM: Nanomolar
µg/mL: Microgram per milliliter
mM: Millimolar
mL: Milliliter
kDa: Kilodalton
µmol/L: Micromole per liter
mg mL^−^^1^: Milligram per milliliter
IC_50_: Half-maximal inhibitory concentration
GI_50_: The concentration for 50% of maximal inhibition of cell proliferation
TGI_50_: Concentration causing 50% growth inhibition
CT_50_: Cytotoxicity titer 50
*K*_is_: Rate of cell growth
SAR: Structure Activity Relationship
